# A Multicomponent Intervention to Reduce Screen Time Among Children Aged 2-5 Years in Chandigarh, North India: Protocol for a Randomized Controlled Trial

**DOI:** 10.2196/24106

**Published:** 2021-02-11

**Authors:** Nimran Kaur, Madhu Gupta, Prahbhjot Malhi, Sandeep Grover

**Affiliations:** 1 Department of Community Medicine and School of Public Health Postgraduate Institute of Medical Education and Research Chandigarh India; 2 Department of Pediatrics Postgraduate Institute of Medical Education and Research Chandigarh India; 3 Department of Psychiatry Postgraduate Institute of Medical Education and Research Chandigarh India

**Keywords:** multimedia, digital-media, preschooler, sedentary behaviors, toddler, sedentary, screen, children, youth

## Abstract

**Background:**

Excessive digital screen exposure (≥1 hour per day) is associated with limited growth and development in children.

**Objective:**

This study aims to develop and assess a multicomponent intervention program's effectiveness in reducing excessive screen time among children aged 2-5 years.

**Methods:**

A theory-based multicomponent intervention known as Program to Lower Unwanted Media Screens (PLUMS) at the household level has been developed. It is based on the social cognitive theory for children and self-determination theory for caregivers. After pretesting, a randomized control trial will be conducted to assess this intervention's effectiveness among healthy children aged 2-5 (±3 months) years and their primary caregivers who have at least one digital media gadget at home in zone three of Chandigarh (population of 2,730,035). A sample size of 428 children is estimated per arm. PLUMS includes disseminating specific information, education, communication in the form of videos and posters to the primary caregivers, and conducting motivational interviewing as and when needed. Children will be provided suggestions for playful activities as alternatives to digital media gadgets. The primary outcome is the mean change in the duration of screen time, and secondary outcomes are sleep duration and patterns, emotional-behavioral problems, and level of physical activity of the children. Per-protocol and intention-to-treat analyses will be conducted using SPSS for Macintosh, Version 25.0.

**Results:**

The intervention package will be disseminated once a week for 8 weeks to the participants via the caregivers' preferred means of communication. The endline assessment will be done immediately postintervention and after the 6 months of follow-up. The Institute's ethics committee, Postgraduate Institute of Medical Education and Research, Chandigarh, India, has approved this study (INT/IEC/2019/000711). The Indian Council of Medical Research, New Delhi (3/1/3/Next-100/JRF-2015/HRD), and PGIMER, Chandigarh (71/2-Edu-16/92, Dated 08/01/2018) funded this study.

**Conclusions:**

PLUMS might be effective in reducing excessive screen time among children aged 2-5 years in a North Indian Union Territory.

**Trial Registration:**

Clinical Trial Registry India CTRI/2017/09/009761; https://tinyurl.com/53q6dpjs

**International Registered Report Identifier (IRRID):**

DERR1-10.2196/24106

## Introduction

### Background

The overall impact of sedentary behaviors on health across the lifespan has gathered widespread recognition, as it plays a significant role in most noncommunicable diseases (NCDs) [1]. The Sustainable Development Goals 2030 [2] envisions reducing premature mortality from NCDs by one-third via the prevention, treatment, and promotion of mental health and well-being. Around 34% to 94% of children aged 2 to 5 years remain sedentary during the day [3]. The prevalence of television (TV) viewing for more than 2 hours per day was reportedly 83% in the United States [4] and up to 82% in Canada [5] in children less than 5 years old and 78% in Australia [6] among children aged 3 to 5 years. A Thai study reported that children aged 1 year viewed TV for 1.23 (SD 1.42) hours per day, which increased to 1.69 (SD 1.56) hours per day when they turned 2 years old [7].

Early childhood is a crucial period that is marked by rapid growth and development [8]. Therefore, it is essential to correct an excessively sedentary lifestyle at an early age. These sedentary pursuits may have specific health consequences during this period and later in life [9]. The health effects of excessive screen time (ST) include behavioral problems; poor language, cognitive development, skills, memory, and executive function; lower social competence; sleep disturbances; depression; and low self-esteem [10-17]. In light of this, the present randomized controlled trial was planned with an assumption that ST might be causally associated with emotional behaviors, sleep behaviors, and children’s level of physical activity. ST of more than 1 hour per day among children aged 2-5 years is deemed excessive as per the American Academy of Pediatrics [18]. Similar guidelines are available in many developed countries (Australia [19], New Zealand [20], France [21], Italy [22], Canada [23], and World Health Organization [24]) for children aged 2-5 years.

### Need for the Trial

A recent review [25] of intervention studies to reduce ST conducted in the last decade (2008-2018) has shown that all the studies (n=16) were conducted in developed countries. Behavior change theories were incorporated in most of the interventions. Educational material was shared with parents with or without their children. Nearly all the significant studies incorporated a postintervention follow-up. Published literature on ST reduction in low- and middle-income countries among young children is scarce [26,27]. Also, there is minimal evidence on the burden, impact, and effectiveness of interventions to reduce ST in Indian settings. Although some of the studies conducted in India have measured change in ST in young children, it was observed only as a secondary outcome [28]. As the number of TV users in India is almost equal to its population [27], designing an intervention to reduce ST among young children becomes more important. Given the Covid-19 pandemic situation, most children’s digital-screen exposure occurs at home as families are stuck at home with these media gadgets. Parents are an essential liaison of change between the child and health care worker [29]. Therefore, there is an urgent need to devise interventions for those of a younger age in the home environment to prevent adverse health consequences later in life.

### Study Objectives

Given this background, the current study aims to develop a Program to Lower Unwanted Media Screens (PLUMS) to reduce ST among children aged 2-5 years and assess the effectiveness of PLUMS on reducing ST among children in Chandigarh, Union Territory, North India. Effectiveness of the intervention will be determined using the percentage risk reduction of mean ST in the intervention group compared to the control group. As excessive ST is a much less emphasized epidemic, this study's results might focus policy makers on this issue and help them formulate guidelines regarding ST use among young children in India and other low- and middle-income countries.

## Methods

The intervention study will be conducted in 2 phases, including the preintervention phase used to develop the intervention and intervention phase, as described in the following sections.

### Phase I. Preintervention Phase

The multidimensional intervention package was designed in 3 stages, including literature review, formative research, and pretesting. All 3 stages were completed. The brief methodology and results of each stage are described in the following sections.

#### Stage I: Review of Literature

In the first stage, the draft PLUMS, including parent and child modules, was prepared after reviewing the existing literature from 2008-2018. Effective community-based interventions to reduce ST in children and the most commonly used theories or models to design the intervention program were identified [25,30]. The following 3 theoretical models were used: social cognitive theory, self-determination theory, and transtheoretical model of behavior change. Social cognitive theory and self-determination theory were used to develop the intervention, and the transtheoretical model of behavior change was used to understand the stage of behavior change of the caregivers, so that the intervention could be customized accordingly. These theoretical models are described in the following paragraphs.

Social cognitive theory was used to modify preschoolers’ cognitive development by developing targeted strategies to modify the behaviors of the caregivers and children regarding ST or the home media environment. Bandura’s social cognitive theory explains how learning occurs in a social context with a reciprocal and dynamic interaction of the person (here child or caregiver), behavior, and environment. This theory supports a causation model that involves triadic reciprocal determinism between behavioral (expectations, goals, and self-perceptions), personal (cognition, outcome expectation, and efficacy expectation), and environmental factors (reinforcement and observational learning). These factors influence each other bidirectionally (Multimedia Appendix 1). When designing the intervention, we used this theory’s framework of modeling (by the caregivers and health care worker), production (alternatives to ST, such as activities given to children or suggested by them), retention (repeated positive feedback provided by a health care worker), and reinforcement (rewards for encouraging positive behavioral outcomes offered by the health care worker) in observational learning, which is pivotal due to the limited cognitive development of preschoolers [18].

Self-determination theory was used to design the strategies to keep the caregivers motivated to limit the ST among the children and modify the home media environment. Self-determination theory [31] targets 3 primary needs: competence, autonomy, and internalization of the learned concepts leading to desired outcomes. This theory postulates that individuals are better motivated if they satisfy these 3 psychological needs, in turn, leading to positive health outcomes. High-quality learning and favorable outcomes have been observed with the combination of self-determination theory and motivational interviewing [32]. The 4 major motivational interviewing components are empathy, developing discrepancy, “rolling with resistance,” and supporting self-efficacy [33]. So, motivational interviewing, along with self-determination theory [34], will be used with the caregivers. Theory-based motivational interviewing will herald the researcher to instruct the caregivers (Multimedia Appendix 2). These motivated and self-determined caregivers will then role-model learned behaviors at home to realize the same in their children and subsequently change the home environment [31].

Last, the transtheoretical model of behavior change by Prochaska et al [33] will be used to modify the caregivers’ behaviors. The stage of change will be identified for each caregiver as per the transtheoretical model (Multimedia Appendix 3). The caregivers will be educated and guided towards the desired behavior, eventually preventing relapse.

#### Stage II: Formative Research

In the second stage, the children’s primary caregivers and trained clinicians suggested workable alternatives to ST for children as a result of the formative research. The ideas gathered were refined by an expert panel.

A qualitative study was conducted among parents of children aged 2-5 years and service providers working in a tertiary care hospital in Chandigarh, India. In-depth interviews were conducted (30-40 minutes) with caregivers (n=20) attending the outpatient department. Two focus group discussions were conducted (40-60 minutes) with the service providers (n=11) at a predecided venue and time. The interviews were conducted in the participant’s preferred language until data saturation using pretested guides (Multimedia Appendix 4). These interviews were audio-recorded with prior informed consent. Subsequently, thematic analysis was done on the transcribed and translated English versions.

To review and modify the activities and methods proposed by caregivers and clinicians, a consultation meeting with 3 experts (professor-level) from psychiatry, child psychology, and public health was conducted. To decelerate excessive and unregulated ST, the clinicians proposed to increase the quality of family time at home with structured parenting approaches. Caregivers should presume responsibility and effectively use leisure time for children and themselves with health care providers’ help when necessary.

The qualitative data obtained through formative research were transcribed and translated into the English language. Thematic analysis of the data was done manually based on grounded theory. Two authors coded the data independently. Both deductive and inductive logic was applied to reason the acceptability and experience of the participants.

The background information of the participants of the in-depth interview and focus group discussions is given in Supplementary Table 1 in Multimedia Appendix 5.

Among the 20 participants in the in-depth interviews, 14 were mothers, and 6 were fathers. The caregivers had a mean age of 34.2 years. Half (10/20, 50%) of the caregivers were educated until the post-graduate level, 50% (10/20) were working, and 40% (8/20) were public health experts. Most (15/20, 75%) of the mothers were the primary caregivers. They were directly involved in their child's behavior modification and introduced media gadgets to them. Digital media exposure usually occurred at home under adult supervision, and aversive measures were frequently used to regulate media gadgets. Most of the caregivers reported negative consequences of excessive ST on their child’s health like a change in sleep patterns, learning bad language, impaired emotional behavior, increase in sedentary behaviors, and deterioration in concentration; however, a few perceived a gain in knowledge and development of skills.

In the focus group discussions, the service providers (n=11) had a mean age of 29 years. They were mostly unmarried (9/11, 82%), female (7/11, 64%), and working as junior residents (6/11, 55%). Of the resident doctors, 7 were from the Department of Pediatrics, and 4 were from psychiatry. The clinicians had learned about managing such cases from research articles, methods taught by their seniors, and practical things that worked for their children. Also, they had adapted themselves to the guidelines from developed countries as none exist in India.

Regarding the intervention for children to reduce ST, they reported that at home, adults should instill healthy habits, develop hobbies, and communicate ill effects of excessive usage to their children using role modeling. At the school level or hospital level, a designated person should advise in a stepwise fashion using face-to-face communications with consistent modes of communication preferred by the participant. The intervention package was adapted as per the results of the qualitative study.

#### Stage III: Intervention: Program to Lower Unwanted Media Screens (PLUMS)

Two modules, one for the caregivers or parents and another for the children, were developed as part of the PLUMS intervention. The strategies were incorporated separately for caregivers and children. These modules have theme-based activities for the children and videos for the parents or caregivers to engage the children in media screen–free activities. PLUMS will be implemented for 8 weeks (2 months) and will consist of 8 themes. Details of these modules are provided in Supplementary Table 2 in Multimedia Appendix 5.

In the caregivers’ module, online videos will be disseminated weekly for 8 weeks via the parent’s preferred mode of communication. We developed the videos in Hindi and the English language. We developed 1 introductory video and 7 small video sessions based upon the weekly theme. Caregivers can go through the session in their own time as per their convenience, except for the introductory video. The introductory video is planned to be watched with the lead author for a better understanding of the caregivers. The introductory session will last 15-20 minutes, and the 7 weekly videos are 5 minutes long. These online video sessions’ primary objective is to help the parents change the home media environment, provide alternatives to ST, provide tips and cues for parenting skills, and engage the child in media-free activities of their choice. These videos will also provide 10 alternative activities per week for children. The caregivers will be encouraged to reward the children for abiding by the rules and accomplishing goals [35].

The child module’s lesson plans were drawn from educational disciplines like skill building, motor coordination, learning, goal setting, music, dance, arts, and crafts. This module contains activities for the child that need the caregiver’s supervision. To capture the child’s interest and channel their energy, specific activities have been selected. This module emphasizes observational learning and increasing the attention span of the child [36]. The curriculum will also allow children to repetitively and effectively learn the desired behavior(s) to facilitate the production and retention processes [36]. We will be providing the following resources as an alternative to screen time for the children: a reading book called “Chika Chika Boom Boom”; pretested and self-developed comic book on the consequences of ST; and coloring book with crayons.

#### Stage IV: Pretesting the Intervention

In the third stage, the PLUMS intervention, with its parent and child modules, was pretested among 10 families to assess the feasibility, acceptability, and adherence to the intervention plan. After the pretesting, the videos’ language was further simplified to match the parents’ understanding. The introductory video’s duration was reduced from 18 minutes to 12 minutes, as suggested by the families. The videos were made attractive by adding animations, bright colors, background music, and live videos of children (regarding behaviors) recommended by the parents. Additional indoor alternatives to ST were included for the children, as parents could not take the child for outdoor play due to Covid-19 pandemic restrictions. The child module now has 10 activities to choose from instead of 1 per day, as was planned earlier. The children will also be given a coloring book with crayons. The intervention was acceptable and feasible, and the caregivers were ready to adhere to the proposed plan.

Finally, the modified intervention module will be used in the main intervention study.

### Phase II: Intervention Study

#### Study Setting

This study will be conducted in Chandigarh, a North Indian Union Territory. It has a population of about 1,055,450 as per the 2011 census [37]. It is divided into 4 zones. This study will be conducted in Zone three, a field practice area of the Department of Community Medicine and School of Public Health, Postgraduate Institute of Medical Education and Research, Chandigarh, India (Multimedia Appendix 6). It has a population of 250,000 as per the 2018-2019 annual health survey. There are approximately 8681 children aged 2-5 years in this area.

#### Study Design

The intervention study is a randomized controlled trial.

#### Study Population

The study population includes the families (N=8681) with children aged 2-5 years (±3 months). The primary caregiver was considered the person who spent most of the time with the child and was involved in the child’s decision making for various activities. Inclusion criteria are families who consent to be part of the study, are residents of the study area in Chandigarh for at least the past 6 months, and who intend to stay in Chandigarh through the intervention and follow-up period. Children previously diagnosed (as per medical records) with long-term or chronic illnesses will be excluded from the study.

#### Sample Size

The sample size for the individual level randomization was estimated using the following formula [38]: n_I_= (σ_1_^2^+σ_2_^2^) (Z_1-α/2_+ Z_1-β_)^2^/Δ^2^, where n = sample size, σ_1_ = standard deviation of the control group and assumed to be 2.09 [39], σ_2_ = standard deviation of the intervention group and assumed to be 1.46 [40], Δ = difference in group means of the average ST and assumed to be 0.54 [39], Z_1-α/2_ = two-sided Z value (Z = 1.96 for the 95% CI), and Z_1-β_ = power (80%). Hence, n_I_ was 170 participants per arm. Considering a 10% attrition rate due to loss to follow-up and 15% nonresponse or refusal rate, the sample size per arm was calculated as 214.

#### Sampling Technique

**Figure 1:**
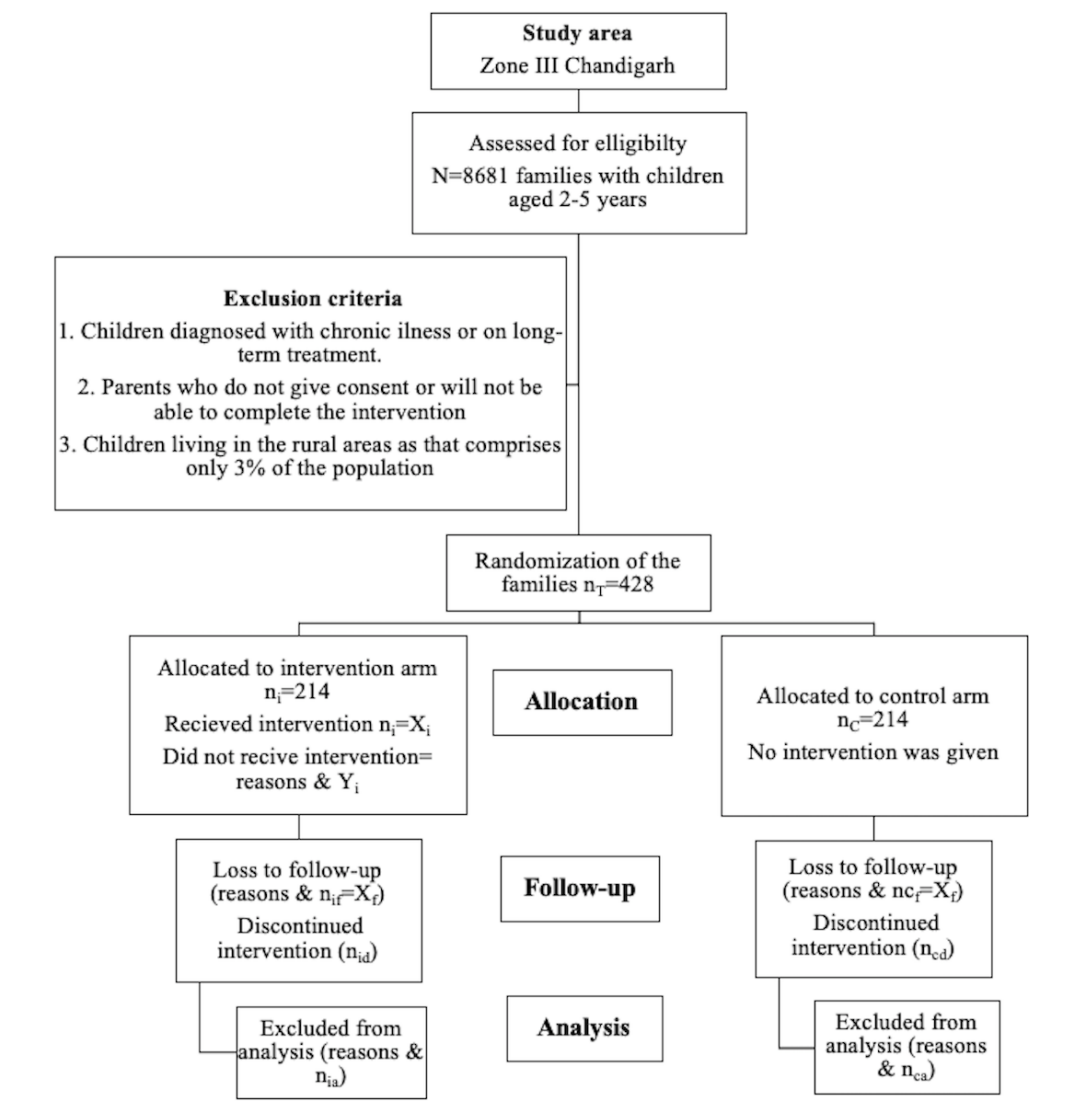
CONSORT statement flow diagram for the intervention study.

The list of eligible families (ie, parents who have children aged 2-5 years) will be obtained from the area’s annual health survey report. Families will be randomly selected using the list obtained from the auxiliary nurse midwife. Eligible families providing consent and willing to complete the intervention and follow-ups will be enrolled in the study and randomized into one of 2 groups: control and intervention. The sampling technique and randomization of families using the Consolidated Standards of Reporting Trials (CONSORT) statement is shown in Figure 1 [41].

#### Randomization

Computer-generated randomization will be done to select families for the intervention and control arms. However, restricted randomization will be done so that intervention and control groups have a similar number of families from high, middle, and low socioeconomic groups. Geographical differences (urban and slum areas) in the study area will also be considered during randomization to avoid contamination. A computer-generated sequence will be used to allocate the families in the intervention and control groups. The person generating the list of sequences, collecting data, and disseminating the intervention will be the same; hence, concealment might not be possible. Moreover, as the intervention package comprises information, education, and communication material, concealment might not be possible. Data collection and delivery of the intervention will be done only after the family’s allocation in the intervention or control arm.

#### Blinding

Blinding will not be possible as the investigator will know the participants in the intervention group because he or she will motivate them to continue the intervention at each step. Also, the participants will know that they are being given an intervention to reduce the ST of their children. However, data entry will be performed by a data entry operator who will be blinded.

#### Stages of the Study

The intervention study will be conducted in 3 stages: baseline assessment, implementation of the intervention, and postintervention assessment.

#### Baseline Assessment

The baseline data were collected from December 2019 to March 2020. During the baseline assessment, the mean ST, prevalence of excessive ST and its correlates, mean duration of physical activity, emotional problems, and sleep disturbances in both the intervention and control arms were measured.

To assess mean ST, physical activity, and prevalence of excessive ST, a pretested, validated, semistructured, bilingual (English and Hindi languages) digital screen exposure questionnaire (DSEQ) was used (Multimedia Appendix 7). The DSEQ was developed in 5 phases. In phase 1, a draft questionnaire was developed by reviewing the literature on existing tools (n=2) from 2009 to 2017. In phase 2, 9 experts assessed the draft Hindi and English questionnaires’ face and content validity. Face-to-face interviews with primary caregivers (n=30) were conducted in a tertiary care hospital for acculturation. In phase 3, a pilot study was conducted among randomly selected families to evaluate the feasibility of the DSEQ in field settings. During phase 4, test-retest reliability was assessed among 30 primary caregivers selected randomly in another urban cluster. In phase 5, the internal consistency of the DSEQ was checked by conducting a cross-sectional study among 400 randomly selected, primary caregivers in Chandigarh, North India. The DSEQ has good internal consistency (Cronbach α=0.73-0.82) and inter-rater agreement (kappa 0.75, 95% CI 0.72-0.78).

The DSEQ was used to collect information from the primary caregivers of the children on (1) sociodemographic profile including family type and socioeconomic status (BG Prasad classification, 2016) [42], (2) digital screen exposure and home media environment, (3) level of physical activity of the child, (4) child’s media-related behaviors, and (5) parental perceptions and literacy regarding digital screen exposure. We recorded the duration and content of media watched by the child on a typical day. The objective criterion to measure ST was the number of hours the child watched a specific TV program. Cinema and movies were excluded from the ST measurement to calculate the actual average of ST for a regular day. Besides, cinema and movies are not watched daily and might falsely increase ST.

We used the 5 progressive physical activity levels among preschoolers given in the Preschool Physical Activity Questionnaire (PrePAQ) to assess physical activity. The PrePAQ categorizes activity into stationery (no action), sedentary (limb or trunk movement), slow-paced (moving easily or slowly), medium-paced (moving at a moderate pace), and fast-paced (quick pace or hard effort) [43].

The standard Preschool Child Behavior Check List was used to assess the child’s emotional and behavioral development. It has been validated in an Indian setting and has the right internal consistency (Cronbach α=0.95) [44]. It consists of 100 items with a 3-step response scale (0, 1, and 2). These items are scored as being absent (score, 0), occasionally present (score, 1), and very often present (score, 2).

The sleep disturbances scale for children was used to measure the child’s sleep patterns. It is a 26-item Likert-type scale that measures specific sleep disorders along with overall sleep disturbances in children. It has been validated and has an internal consistency of 0.71-0.79, test-retest reliability of 0.71, and diagnostic accuracy of 0.91 [40].

The lead author visited the homes of the study families and conducted face-to-face interviews during the baseline assessment.

#### Implementing the Intervention

In this stage, the PLUMS intervention will be delivered over an 8-week period at the household level in the intervention arm, as already described (Supplementary Table 2 in Multimedia Appendix 5). The objectives of the intervention are to reduce the ST of children [18], enable the caregivers with positive parenting skills and ST literacy, and propose unplugged family time in the home environment.

Participants in the intervention arm will also be classified into 5 behavior stages of change as per the transtheoretical model [45]. In the control arm, routine home-based and facility-based health services will be continued by the local auxiliary nurse midwife under the public health system. Compliance of the intervention will be checked via a proforma from parents (Supplementary Table 3 in Multimedia Appendix 5) and via a journal, videos, or pictures maintained by families.

#### Postintervention Assessment

In this stage, the same questionnaire for ST and its correlates as used in the baseline assessment will be administered to the intervention and control groups immediately and 6 months postintervention. Hence, primary and secondary outcome measures will be assessed at these 2 time points. In addition to the 2 months of active intervention, the researcher will maintain contact with the intervention arm group participants during the 6-month follow-up period. A passive intervention via information education material (text messages, telephone, emails, or mail) to parents will be given during the follow-up period.

This intervention study will take place over a period of 8 months. The baseline data collection will take place before the intervention (t_0_). The postintervention phase (t_1_) and follow-up assessment will be done at 6 months (t_2_), as shown in Supplementary Table 4 in Multimedia Appendix 5, as per the Standard Protocol Items: Recommendations for Interventional Trials (SPIRIT) [46].

#### Outcomes

The primary outcome (caregiver-reported) measure will be the mean change in ST (in minutes) per day separately for weekdays and weekends. The secondary outcomes (caregiver-reported) will be the mean change in the duration of physical activity (in minutes), proportion of children with emotional behavior problems, and sleep disturbances. For emotional behavior and sleep disturbances, the change in scores as per the tools will be noted, as mentioned earlier.

#### Data Management and Analysis

Quantitative data from the intervention study will be entered into Microsoft Excel and analyzed using SPSS for Macintosh, Version 25.0 (IBM Corp, Armonk, NY). ST on a weekday and weekend day will be calculated separately as per available literature [47]. So, the average ST (by viewing any type of media gadget) per day in a week will be calculated, and because there are 5 weekdays (Monday to Friday) and 2 weekend days (Saturday and Sunday) in a week, the weighted average ST per day in a week will be calculated as: Average ST per day (minutes) = [(Weekday ST in minutes x 5) + (Weekend ST in minutes x 2)]/7.

Continuous variables will be summarized as means with interquartile ranges and standard deviations. Correlations between a continuous variable and other variables will be estimated. Categorical variables will be summarized as proportions, and differences between groups will be tested using chi square tests. The data will be analyzed as per intention-to-treat analysis (for their initially assigned study arm) and per-protocol analysis. Relative risks and their effect sizes, together with the corresponding 95% CIs, will be estimated. The effectiveness of the intervention will be estimated by calculating the relative risk reduction (1-relative risk). A *P* value <.05 will be considered statistically significant for all analyses.

Information regarding PLUMS will be shared in the first communication and then reinforced every week for 8 weeks. So, participants who withdraw from the trial after the third week will be included in the analysis. Participants who withdraw any time before the third week will be excluded from the analysis.

Caregivers’ knowledge will be analyzed at the end of the intervention if no associations are observed. The proforma on behavior change will be completed at baseline (t_0_) and endline (t_1_) to determine the stage of change the families are at postintervention.

A feedback/compliance questionnaire (Supplementary Table 3 in Multimedia Appendix 5) will be given to the caregivers at each counseling session (weekly) to check their fidelity. The child's fidelity, including the level of engagement, will be checked with personalized journals comprising the activities they performed during the given intervention. The parents will be contacted via their preferred mode of communication weekly to keep them motivated and disseminate the next week's intervention module.

#### Ethics and Dissemination

Ethical approval was obtained from the institutes' ethics committee: Postgraduate Institute of Medical Education and Research, Chandigarh, India (INT/IEC/2019/000711, Dated 02/04/2019).

## Results

Enrollment of all participants (n=440) was completed during the baseline assessment. The intervention phase could not be started due to restrictions imposed under lockdown to contain the Covid-19 pandemic. Considering the Covid-19 pandemic and likely change in the ST and physical activity of the children, we will measure the ST and physical activity again before delivering the intervention. The intervention was started in November 2020. It will be implemented for 8 weeks. The endline assessment will be done immediately postintervention and after the 6 months of follow-up. The results will most likely be published by 2022.

## Discussion

### Principal Findings

Developing countries have seen a recent explosion in digital media, leading to increased ST and sedentary behavior in the younger generation [47]. However, the exact burden and impact of excessive ST exposure, especially among young children, are not well known. An Indian study concluded that 60% of the children (2-5 years) in North India used digital media gadgets for more than 1 hour per day. Bansal et al [48] from South India reported a 72% prevalence of ST for more than 1 hour per day (mobile usage only) among children younger than 15 years [48]. In addition, Indian children (3-11 years old) [49] and Korean children (2-5 years old) [47] experience ST of <1.4 hours per day and <1.21 hours per day, respectively. Since few local studies were available, the present study was planned to reduce the average mean duration of ST in children rather than the proportion.

Previous studies [38] have focused on the usage and impact of TV viewing in children; however, this study includes all digital media types being used by young children (3-5 years old). A systematic review suggested that the most effective interventions for ST reduction in children (0-5 years old) were ≥6 months in duration and conducted in a community setting [30]. Hence, in this study, a home-based intervention was developed to generate evidence on its effectiveness in reducing ST among young children from developing countries. This intervention includes providing feasible alternatives to ST and spending quality family time to strengthen parent-child bonding. Engaging children in goal setting [50] to decrease ST has been proven an effective measure. Also, the intervention plans to design workable measures to limit ST and improve the parent-child relationship in low- and middle-income countries. Further, involving children in alternative activities channels their energy, boosts their confidence, and decreases ST. Since there is evidence that there is a significantly higher risk of abnormal sleep with a TV in the bedroom [50], the interventions in this study include advising caregivers about changing the home media environment to decrease overall ST for children.

With advancements in technology, many newer media gadgets have been introduced in the younger generation’s digital-media ecosystem [51]. The existing literature suggests that sedentary behaviors due to excessive media gadget use in children may adversely affect their overall health and social outcomes; however, the pros and cons of their use have not been well understood [52]. Given this, the present study was planned as a home-based intervention study over 6 months to reduce ST among young children. Randomized controlled trials are a punctilious way of determining a cause-effect relation between treatment and outcome [53]. The present study will adhere to the latest American Academy of Pediatrics guidelines [18], which state that children aged 2-5 years should not be exposed to more than 1 hour of high-quality educational ST. To ensure the participants’ compliance with PLUMS, the intervention plan is intended to be delivered at a place convenient to the participants. The caregivers will be counseled at the time and place of their choice.

### Strengths

The strengths of this study are that the participants represent Chandigarh's population; hence, the study results can be generalized. There are no published intervention studies from India in this age group and on ST [25]. This study will be the first study that provides a home-based intervention plan to families based upon commonly used theories (transtheoretical model of behavior change [33], social cognitive theory [54], and self-determination theory [25]). This intervention complies with the latest American Academy of Pediatrics guidelines (2016), while previous studies were based on older guidelines. Also, this intervention study focuses on enhancing parents’ literacy concerning ST and will eventually have a long-term effect.

### Limitations

Social desirability bias might result in caretakers changing some responses. Last, as there is no reliable measure of child ST, recall bias might be introduced because we are relying on the parents’ memory. However, to overcome these biases, the investigator recorded all the programs the child watched in the last week and the approximate duration of these programs. Then, to prevent recall bias, TV diaries were proposed to capture the children’s actual ST during the day. We will develop a journal comprised of daily activities for children. This journal will cross-check compliance with the intervention and activities preferred by the children at home. Last, there are limited validated tools in literature, so the DSEQ was developed, validated, and pretested to measure ST.

### Conclusion

The results from this study might guide policymakers to formulate guidelines on preventing excessive digital screen exposure among young children, the media content watched by children, and the importance of ST on growth and development in childhood, especially in the context of low- and middle-income countries. The results will also facilitate the implementation of an ST reduction intervention program among children aged 2-5 years, which may help prevent NCDs and the attainment of sustainable development goals.

## Appendix

Multimedia Appendix 1 Social Cognitive Theory (SCT) for designing the intervention.

Multimedia Appendix 2 Self-determination theory for motivational interviewing.

Multimedia Appendix 3 Transtheoretical model for stages of change.

Multimedia Appendix 4 Qualitative interview guides.

Multimedia Appendix 5 Supplementary tables.

Multimedia Appendix 6 Map of Chandigarh showing the study area.

Multimedia Appendix 7 Digital Screen Exposure Questionnaire-DSEQ.

Multimedia Appendix 8 Peer-review reports from the PGIMER Doctoral Committee.

Multimedia Appendix 9 CONSORT 2010 checklist of information to include when reporting a randomized trial.

## Abbreviations

CONSORT: Consolidated standards of reporting trials statement
DSEQ: Digital Screen Exposure Questionnaire
NCD: noncommunicable disease
PLUMS: Program to Lower Unwanted Media Screens
SPIRIT: Standard Protocol Items: Recommendations for Interventional Trials
ST: screen time
TV: television

## References

[1] LeBlanc AG, Spence JC, Carson V, Connor Gorber S, Dillman C, Janssen I, Kho ME, Stearns JA, Timmons BW, Tremblay MS (2012). Systematic review of sedentary behaviour and health indicators in the early years (aged 0-4 years). Appl Physiol Nutr Metab.

[2] (2021). Management of substance abuse; Process of translation and adaptation of instruments. World Health Organization.

[3] Hnatiuk JA, Salmon J, Hinkley T, Okely AD, Trost S (2014). A review of preschool children's physical activity and sedentary time using objective measures. Am J Prev Med.

[4] Tandon PS, Zhou C, Lozano P, Christakis DA (2011). Preschoolers' total daily screen time at home and by type of child care. J Pediatr.

[5] Carson V, Spence JC, Cutumisu N, Cargill L (2010). Association between neighborhood socioeconomic status and screen time among pre-school children: a cross-sectional study. BMC Public Health.

[6] Hinkley T, Salmon J, Okely AD, Crawford D, Hesketh K (2012). Preschoolers' physical activity, screen time, and compliance with recommendations. Med Sci Sports Exerc.

[7] Ruangdaraganon N, Chuthapisith J, Mo-suwan L, Kriweradechachai S, Udomsubpayakul U, Choprapawon C (2009). Television viewing in Thai infants and toddlers: impacts to language development and parental perceptions. BMC Pediatr.

[8] Chief Medical Officer of Health (2011). Let's talk about the early years: early childhood development. Government of Alberta.

[9] Christakis DA, Gilkerson J, Richards JA, Zimmerman FJ, Garrison MM, Xu D, Gray S, Yapanel U (2009). Audible television and decreased adult words, infant vocalizations, and conversational turns: a population-based study. Arch Pediatr Adolesc Med.

[10] Nuutinen T, Ray C, Roos E (2013). Do computer use, TV viewing, and the presence of the media in the bedroom predict school-aged children's sleep habits in a longitudinal study?. BMC Public Health.

[11] Costello EJ, Mustillo S, Erkanli A, Keeler G, Angold A (2003). Prevalence and development of psychiatric disorders in childhood and adolescence. Arch Gen Psychiatry.

[12] Lobo YB, Winsler A (2006). The Effects of a Creative Dance and Movement Program on the Social Competence of Head Start Preschoolers. Social Development.

[13] Garon N, Bryson SE, Smith IM (2008). Executive function in preschoolers: a review using an integrative framework. Psychol Bull.

[14] Bauer P, Larkina M, Deocampo J, Goswami U (2010). Early Memory Development. The Wiley-Blackwell Handbook of Childhood Cognitive Development, Second edition.

[15] Collins ML, Nelson CA (2008). Handbook of developmental cognitive neuroscience, 2 ed. 2nd ed. Nelson CA, Luciana M. editors.

[16] Cardon GM, De Bourdeaudhuij IMM (2008). Are Preschool Children Active Enough? Objectively Measured Physical Activity Levels. Research Quarterly for Exercise and Sport.

[17] Mistry S, Puthussery S (2015). Risk factors of overweight and obesity in childhood and adolescence in South Asian countries: a systematic review of the evidence. Public Health.

[18] Reid Chassiakos YL, Radesky J, Christakis D, Moreno MA, Cross C, Council on Communications and Media (2016). Children and Adolescents and Digital Media. Pediatrics.

[19] (2019). Australia's Physical Activity and Sedentary Behaviour Guidelines and the Australian 24-Hour Movement Guidelines. Australian Government The Department of Health.

[20] Sigman A (2015). We need to talk: screen time in New Zealand. Family First.

[21] Alleyne R (2008). France bans marketing television programmes targeted at under threes. The Telegraph.

[22] Bozzola E, Spina G, Ruggiero M, Memo L, Agostiniani R, Bozzola M, Corsello G, Villani A (2018). Media devices in pre-school children: the recommendations of the Italian pediatric society. Ital J Pediatr.

[23] Tremblay MS, Leblanc AG, Carson V, Choquette L, Connor GS, Dillman C, Duggan M, Gordon MJ, Hicks A, Janssen I, Kho ME, Latimer-Cheung AE, Leblanc C, Murumets K, Okely AD, Reilly JJ, Stearns JA, Timmons BW, Spence JC, Canadian Society for Exercise Physiology (2012). Canadian Sedentary Behaviour Guidelines for the Early Years (aged 0-4 years). Appl Physiol Nutr Metab.

[24] Keenan A (2019). To grow up healthy, children need to sit less and play more. World Health Organization.

[25] Kaur N, Gupta M, Malhi P, Grover S (2019). Screen Time in Under-five Children. Indian Pediatr.

[26] Davey S, Davey A (2014). Assessment of Smartphone Addiction in Indian Adolescents: A Mixed Method Study by Systematic-review and Meta-analysis Approach. Int J Prev Med.

[27] (2018). Basic Statistics of U.T. Chandigarh in Nutshell. Chandigarh Administration.

[28] Thakur JS, Bharti B, Tripathy JP, Dhawan V, Bhansali A (2016). Impact of 20 Week Lifestyle Intervention Package on Anthropometric Biochemical and Behavioral Characteristics of Schoolchildren in North India. J Trop Pediatr.

[29] Dietz WH, Gortmaker SL (1985). Do we fatten our children at the television set? Obesity and television viewing in children and adolescents. Pediatrics.

[30] Downing KL, Hnatiuk JA, Hinkley T, Salmon J, Hesketh KD (2016). Interventions to reduce sedentary behaviour in 0–5-year-olds: a systematic review and meta-analysis of randomised controlled trials. Br J Sports Med.

[31] Deci E, Ryan R, Vallerand R, Pelletier L (1991). Motivation and Education: The Self-Determination Perspective. Educational Psychologist.

[32] Miller LS, Gramzow RH (2016). A self-determination theory and motivational interviewing intervention to decrease racial/ethnic disparities in physical activity: rationale and design. BMC Public Health.

[33] Prochaska JO, Velicer WF, Rossi JS, Goldstein MG, Marcus BH, Rakowski W, Fiore C, Harlow LL, Redding CA, Rosenbloom D, Rossi SR (1994). Stages of change and decisional balance for 12 problem behaviors. Health Psychology.

[34] Markland D, Ryan RM, Tobin VJ, Rollnick S (2005). Motivational Interviewing and Self–Determination Theory. Journal of Social and Clinical Psychology.

[35] Anderson DR, Collins PA (1988). The Impact on Children's Education: Television's Influence on Cognitive Development. Working Paper No 2.

[36] Mendoza JA, Baranowski T, Jaramillo S, Fesinmeyer MD, Haaland W, Thompson D, Nicklas TA (2016). Fit 5 Kids TV Reduction Program for Latino Preschoolers: A Cluster Randomized Controlled Trial. Am J Prev Med.

[37] Chandramouli C (2011). Census of India 2011: Provisional Population Totals. Indian Administrative Service.

[38] Teare MD, Dimairo M, Shephard N, Hayman A, Whitehead A, Walters SJ (2014). Sample size requirements to estimate key design parameters from external pilot randomised controlled trials: a simulation study. Trials.

[39] Haines J, McDonald J, O'Brien A, Sherry B, Bottino CJ, Schmidt ME, Taveras EM (2013). Healthy Habits, Happy Homes: randomized trial to improve household routines for obesity prevention among preschool-aged children. JAMA Pediatr.

[40] Bruni O, Ottaviano S, Guidetti V, Romoli M, Innocenzi M, Cortesi F, Giannotti F (1996). The Sleep Disturbance Scale for Children (SDSC). Construction and validation of an instrument to evaluate sleep disturbances in childhood and adolescence. J Sleep Res.

[41] Pandis N, Chung B, Scherer RW, Elbourne D, Altman DG (2017). CONSORT 2010 statement: extension checklist for reporting within person randomised trials. BMJ.

[42] Pandey VK, Aggarwal P, Kakkar R (2018). Modified BG Prasads Socio-economic Classification-2018: The need of an update in the present scenario. Indian Journal of Community Health.

[43] Dwyer GM, Hardy LL, Peat JK, Baur LA (2011). The validity and reliability of a home environment preschool-age physical activity questionnaire (Pre-PAQ). Int J Behav Nutr Phys Act.

[44] Basu B, Dutta N (2010). Psychological changes of children surviving terrorist shock in Indian Kashmir. J Child Neurol.

[45] Hashemzadeh M, Rahimi A, Zare-Farashbandi F, Alavi-Naeini A, Daei A (2019). Transtheoretical model of health behavioral change: A systematic review. Iranian J Nursing Midwifery Res.

[46] Chan A, Tetzlaff JM, Gøtzsche PC, Altman DG, Mann H, Berlin JA, Dickersin K, Hróbjartsson A, Schulz KF, Parulekar WR, Krleza-Jeric K, Laupacis A, Moher D (2013). SPIRIT 2013 explanation and elaboration: guidance for protocols of clinical trials. BMJ.

[47] Chang HY, Park E, Yoo H, Lee JW, Shin Y (2018). Electronic Media Exposure and Use among Toddlers. Psychiatry Investig.

[48] Bansal S, Mahajan RC (2017). Impact of mobile use amongst children in rural area of Marathwada region of Maharashtra, India. Int J Contemp Pediatr.

[49] Gulati A, Hochdorn A, Paramesh H, Paramesh EC, Chiffi D, Kumar M, Gregori D, Baldi I (2014). Physical activity patterns among school children in India. Indian J Pediatr.

[50] Radesky JS, Christakis DA (2016). Increased Screen Time: Implications for Early Childhood Development and Behavior. Pediatr Clin North Am.

[51] Bandura A (1987). Social Foundations of Thought and Action: A Social-Cognitive View. AMR.

[52] (2016). AAP Announces New Recommendations for Children’s Media Use. American American Academy of Pediatrics.

[53] Sibbald B, Roland M (1998). Understanding controlled trials. Why are randomised controlled trials important?. BMJ.

[54] Bandura A (1978). Social learning theory of aggression. J Commun.

