# Supplementary figures and images for: A Multicomponent Intervention to Reduce Screen Time Among Children Aged 2-5 Years in Chandigarh, North India: Protocol for a Randomized Controlled Trial
Source: JMIR Res Protoc. 2021 Feb 11;10(2):e24106. doi: 10.2196/24106 (PMC7906833; doi:10.2196/24106)

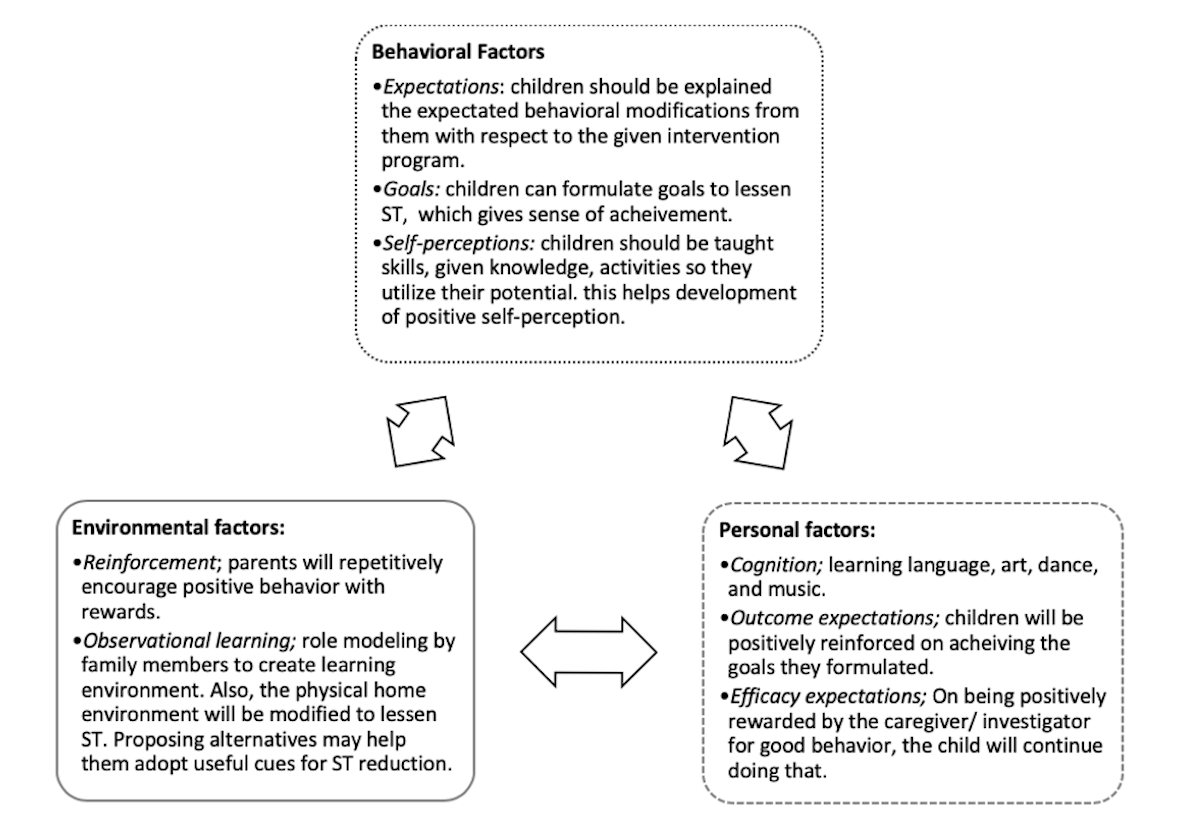

Supplement: Multimedia Appendix 1 [file resprot_v10i2e24106_app1.png]

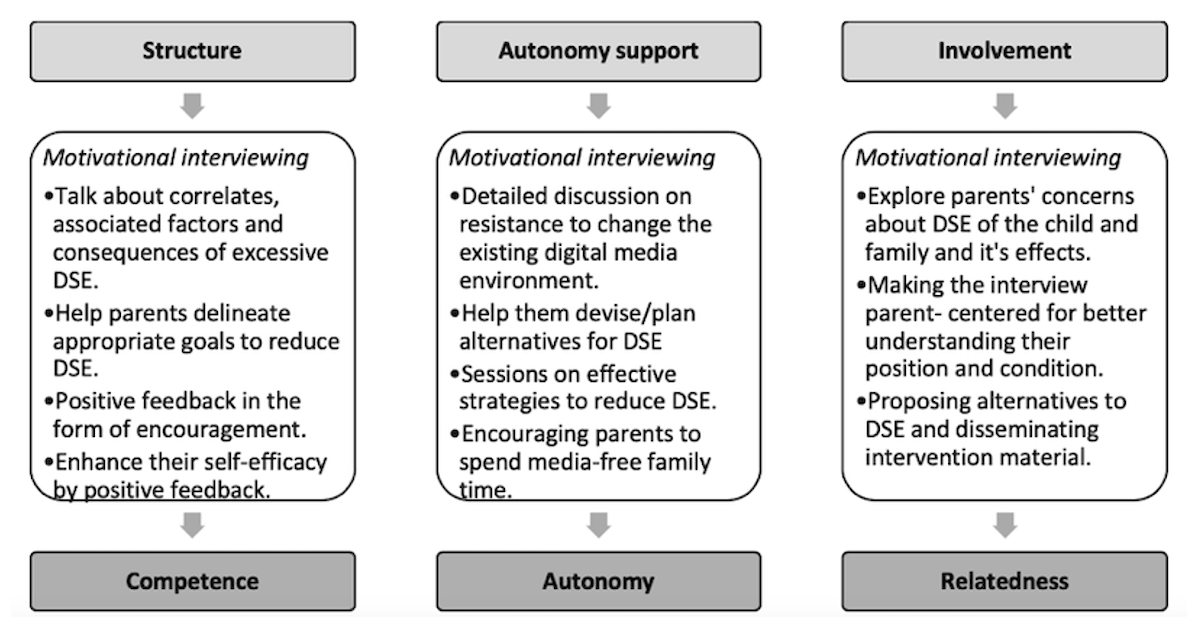

Supplement: Multimedia Appendix 2 [file resprot_v10i2e24106_app2.png]

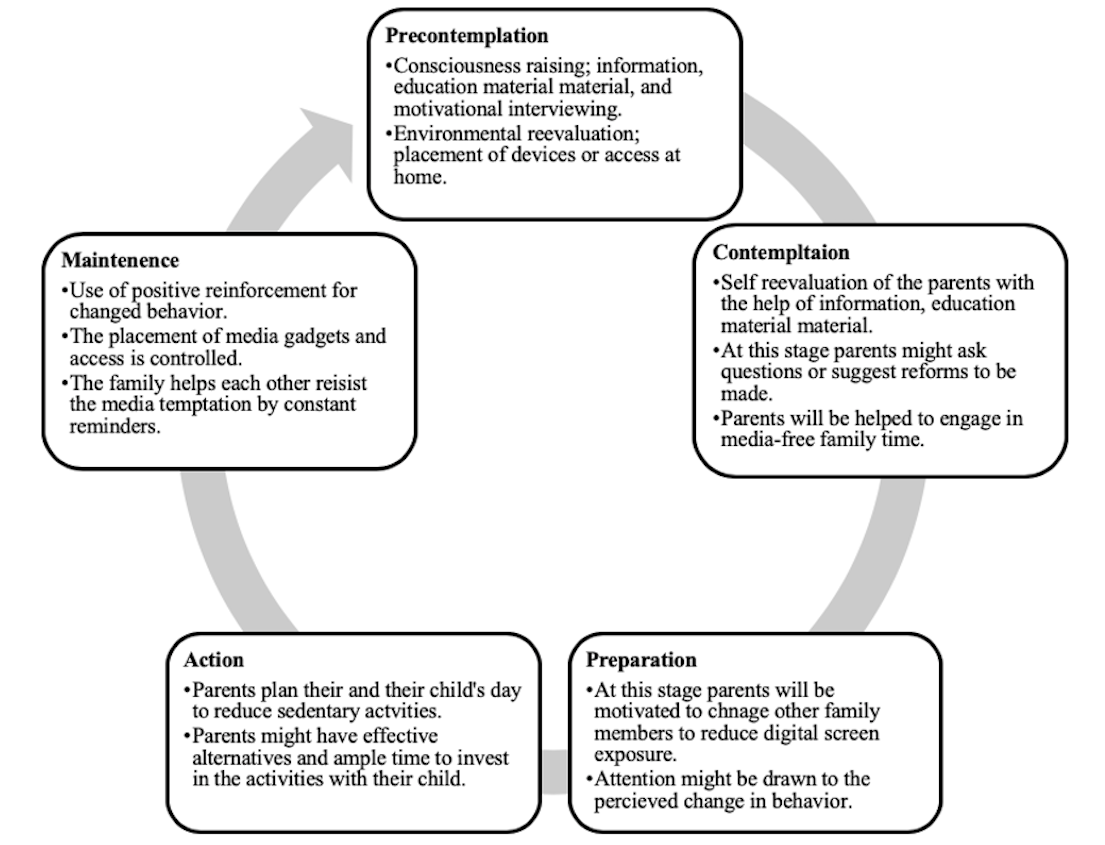

Supplement: Multimedia Appendix 3 [file resprot_v10i2e24106_app3.png]

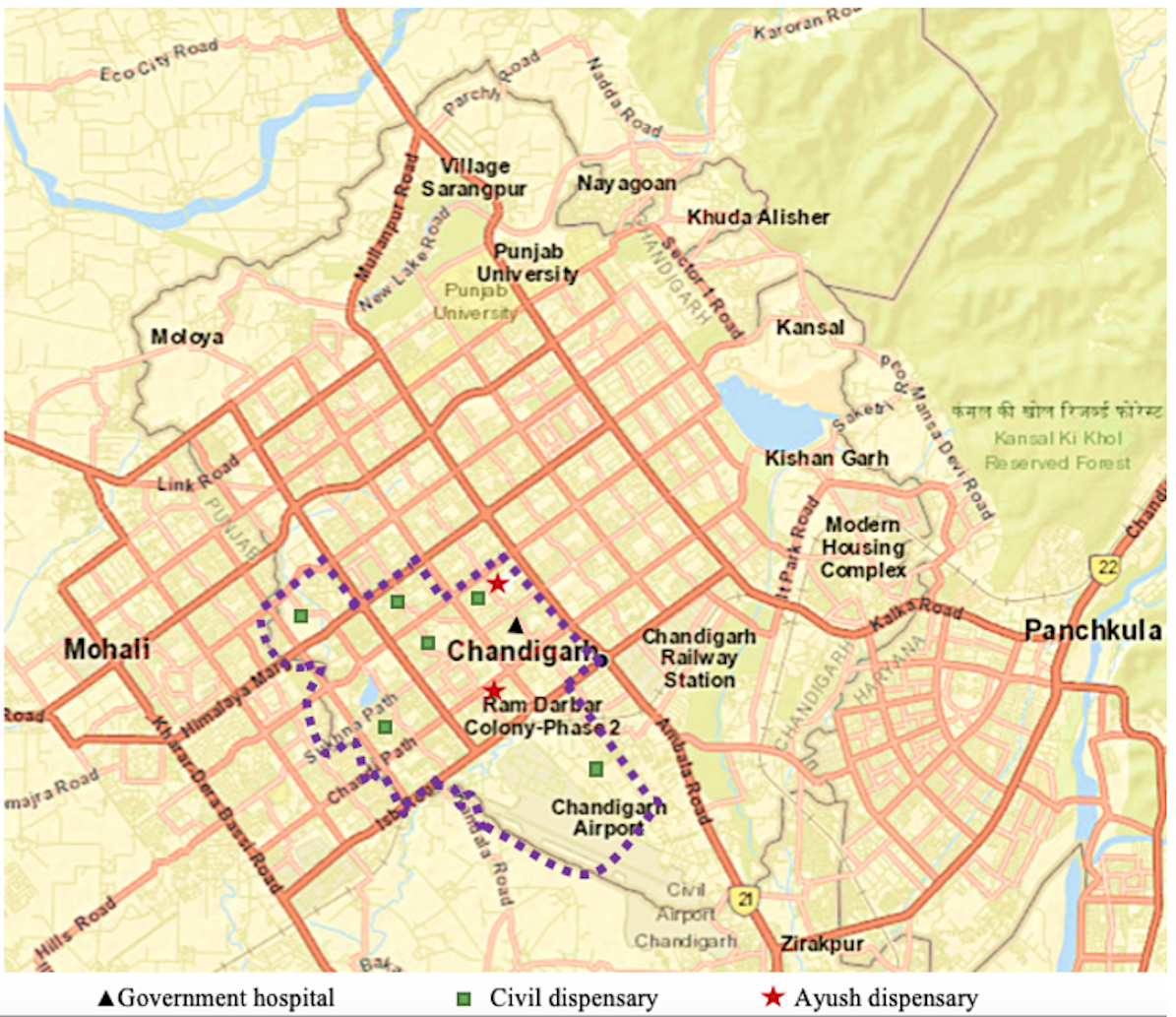

Supplement: Multimedia Appendix 6 [file resprot_v10i2e24106_app6.png]
