# Supplementary material for: A Multicomponent Intervention to Reduce Screen Time Among Children Aged 2-5 Years in Chandigarh, North India: Protocol for a Randomized Controlled Trial
Source: JMIR Res Protoc. 2021 Feb 11;10(2):e24106. doi: 10.2196/24106 (PMC7906833; doi:10.2196/24106)
Supplement: Multimedia Appendix 4 [file resprot_v10i2e24106_app4.docx]

**Supplementary File 2. Qualitative interview guides.**

1. **In-depth Interview (IDI) Guide with the caregivers/parents**

**Section 1. Informed Consent**

**Section 2. Background information of the participants**

1. Unique ID:
2. Name:
3. Age in years:
4. Sex:
5. Relationship to the child:
6. Education level:
7. Occupation:
8. Monthly income:
9. Marital status:
10. Working status:

| **Section 3. IDI Guide** |
| --- |
| 1. **Introduction**     1. Who takes care of the child at home?    2. Do you know digital screen exposure (DSE) can be excessive?    3. Why do you think your children remains engaged to digital media devices? 2. **Consequences of DSE**    1. How do you think DSE can affect your child; positively and negatively?    2. Do you think DSE is affecting the level of activity of your child?       1. What do you do to improve his level of activity?       2. Would you like to enhance your child’s level of physical activity?    3. Do you think DSE is causing sleep disturbances in your children?       1. What do you do about to make your child to go to sleep?       2. Would you like me to suggest you something about it?    4. Do you think DSE can cause any behavioural changes in children?       1. What do you do about your child’s behavioural problems?       2. Would you like to do something about your child’s behaviour?    5. Do you feel DSE is hampering your bond with your child? 3. **Alternatives to DSE**    1. How do you think DSE can be reduced?    2. What alternatives do you suggest?    3. Have you tried these alternatives on your child?    4. Does the child like these suggested activities?    5. Do you think your child would require age-specific alternatives? Or does he paint, draw, read (planned intervention strategies)? 4. **Implementation of DSE alternatives**    1. Do you feel accessibility to digital-media devices might be a problem at home?    2. Do you think you change this at your home?    3. Do you feel change in placement of TV or replacing smartphone with other alternative activities might reduce this DSE in your child?    4. Do you think an intervention can be planned to reduce DSE?    5. How will you like to receive this intervention?    6. What would be the average time that you can spend on the child to reduce DSE in a day?    7. How frequently would you like to receive reminders regarding the intervention? 5. **Intervention**     1. Highlight anything that could keep the family and the child motivated throughout the intervention?    2. Who do think is the role-model to your child regarding DSE habits?    3. Do you think we can convince him/ her (role-model) to change? |

1. **Focus group discussion (FGD) guide with the service providers**

**Section 1. Informed Consent**

**Section 2. Background information of the participants**

1. Unique ID:
2. Name:
3. Age in years:
4. Sex:
5. Education level:
6. Occupation:
7. Monthly income:
8. Marital status:
9. Working status:

| **Section 3***.* **FGD Guide** |
| --- |
| 1. Do parents come to the clinic complaining of excessive digital media exposure in their children? 2. How can we give the clinicians the relevant parenting tips? 3. What feasible strategies have the clinicians tried in your clinics or homes? 4. How can we motivate parents to talk to their children and involve them in decision making with respect to digital media at home? 5. Which out of the suggested alternatives best appealed you? 6. What all consequences have you come across in your clinics? 7. Do parents read the given instructions? Or verbally explaining them is better? 8. Do you know of any guidelines that you can suggest for screen-time that I can adhere to? 9. Do you think the intervention plan needs to be age or gender specific for children? 10. Is it normal for children to have multiple role-models? |
