# Supplementary material for: A Multicomponent Intervention to Reduce Screen Time Among Children Aged 2-5 Years in Chandigarh, North India: Protocol for a Randomized Controlled Trial
Source: JMIR Res Protoc. 2021 Feb 11;10(2):e24106. doi: 10.2196/24106 (PMC7906833; doi:10.2196/24106)
Supplement: Multimedia Appendix 5 [file resprot_v10i2e24106_app5.docx]

**Supplementary Table 1. Socio-demographic characteristics of the study participants of focus group discussion and in-depth interview.**

| Qualitative Approach | In-depth Interviews | | Focus Group Discussion | |
| --- | --- | --- | --- | --- |
| **Study Participants** | **Parents (N=20)** | | **Clinicians (N=11)** | |
| **Characteristics** |  |  |  |  |
| Average age in years | 34.2 | - | 29 | - |
| Average monthly income in USD | 638 |  | 1073 |  |
|  | **Number** | **Percentage** | **Number** | **Percentage** |
| **Sex** |  |  |  |  |
| Male | 5 | 25 | 4 | 36.4 |
| Female | 14 | 70 | 7 | 63.6 |
| **Marital status** |  |  |  |  |
| Yes | 20 | 100 | 2 | 0.18 |
| No |  |  |  |  |
| **Relationship to the child** |  |  |  |  |
| Fathers | 6 | 30 | 1 | 9 |
| Mothers | 14 | 70 | 1 | 9 |
| **Education** |  |  |  |  |
| Below master’s degree | 10 | 50 | 1 | 9 |
| Above master’s degree | 10 | 50 | 10 | 90.9 |
| **Occupation** |  |  |  |  |
| Senior resident doctor | - | - | 4 | 34.4 |
| Junior resident doctor | - | - | 7 | 65.6 |
| Public health expert | 8 | 40 | - | - |
| Others (clerk/security-guard/lab technician/field investigator) | 7 | 35 | - | - |
| Unemployed | 5 | 25 | - | - |
| **Working status** |  |  |  |  |
| Working fathers | 20 | 100 | - | - |
| Working mothers | 15 | 75 | - | - |
| **Monthly income in USD** |  |  |  |  |
| <608 | 13 | 65 | 1 | 9 |
| >608 | 7 | 35 | 10 | 90.9 |

**Supplementary Table 2. Intervention plan for caregivers and children**

| Topic  *Week* | Parent module  *Session* | Time required | Weekly assessment | Child Module  *Activities* | Time required | Weekly assessment |
| --- | --- | --- | --- | --- | --- | --- |
| Growing up without screens is fun (Milestones)  *Week 1* | Excessive screen-time and development milestones of children | 30 minutes | Compliance checklist | Weekly activities, and CDC milestones book (Amazing Me) | 30 - 60 minutes | Activity booklet |
| Material | Video and counselling |  | Supplementary Table 3 | Book 1, Video |  | Video |
| Screens away (Media rules)  *Week 2* | Screen-time rules at home | 30 minutes | Compliance checklist | Weekly activities | 30 - 60 minutes | Activity booklet |
| *Material* | Video and counselling |  | Supplementary Table 3 | Video |  | Journal |
| Home sweet home (Media rules)  *Week 3* | Home media environment | 30 minutes | Compliance checklist | Weekly activities | 30 - 60 minutes | Activity booklet |
| *Material* | Video and counselling |  | Supplementary Table 3 | Video |  | Journal |
| Sound sleeper (Media Rules)  *Week 4* | Sleep and digital media | 30 minutes | Compliance checklist | Weekly activities, and Comic book | 30 - 60 minutes | Activity booklet |
| *Material* | Video and counselling |  | Supplementary Table 3 | Book 2, Video |  | Journal |
| Chatter box  *Week 5* | Effective Communication within family | 30 minutes | Compliance checklist | Weekly activities | 30 - 60 minutes | Activity booklet |
| *Material* | Video and counselling |  | Supplementary Table 3 | Video |  | Journal |
| Hungry for food not media (Media rules)  *Week 6* | Meal time and digital media gadgets | 30 minutes | Compliance checklist | Weekly activities | 30 - 60 minutes | Activity booklet |
| *Material* | Video and counselling |  | Supplementary Table 3 | Video |  | Journal |
| Media rules  *Week 7* | Education in positive experiences in a community setting | 30 minutes | Compliance checklist | Weekly activities | 30 - 60 minutes | Activity booklet |
| Material | Video |  | Supplementary Table 3 | Video |  | Journal |
| Reinforce (building bonds)  *Week 8* | Positive reinforcement and counselling | 30 minutes | Compliance checklist | Weekly activities, and activity booklet | 30 - 60 minutes | Activity booklet |
| *Material* | Video and counselling |  | Supplementary Table 3 | Video |  | Journal |

**Supplementary Table 3. Overview process for evaluation and scoring of the intervention package for parents. (Total score=40).**

| **Package delivered** | | **Package received-exposed** | **Level of understanding-satisfaction** |
| --- | --- | --- | --- |
| (1-32) Did you receive the following information education communication material:  Mostly/Often (2); Sometimes (1); Never (0) | | 33. How frequently did you implement the activities suggested?  Mostly/Often (2); Sometimes (1); Never (0)  34. Did you change anything at home suggested by the IEC material?  M; not always; Nothing  35. How frequently did you spend the suggested time duration for these activities?  Mostly/Often (2); Sometimes (1); Never (0) | 36. In general, how easy were the activities to deliver?  Mostly/Often (2); Sometimes (1); Never (0)  37. In general, did you find the information trustful?  Mostly/Often (2); Sometimes (1); Never (0)  38. Did you easily understand the text?  Mostly/Often (2); Sometimes (1); Never (0)  39. Did you like the design of the IEC material (colours, animations, lay-out, type of letters)  Mostly/Often (2); Sometimes (1); Never (0)  40. Did you find these activities useful?  Mostly/Often (2); Sometimes (1); Never (0) |
| 1. IEC for parents week 1 |  |  |  |
| 1. IEC for parents week 2 |  |  |  |
| 1. IEC for parents week 3 |  |  |  |
| 1. IEC for parents week 4 |  |  |  |
| 1. IEC for parents week 5 |  |  |  |
| 1. IEC for parents week 6 |  |  |  |
| 1. IEC for parents week 7 |  |  |  |
| 1. IEC for parents week 8 |  |  |  |
| 1. Activity for child week 1 |  |  |  |
| 1. Activity for child week 2 |  |  |  |
| 1. Activity for child week 3 |  |  |  |
| 1. Activity for child week 4 |  |  |  |
| 1. Activity for child week 5 |  |  |  |
| 1. Activity for child week 6 |  |  |  |
| 1. Activity for child week 7 |  |  |  |
| 1. Activity for child week 8 |  |  |  |
| (18-34) Did you or your spouse read the information education communication material  Mostly/Often (2); Sometimes (1); Never (0) | |  |  |
| 1. IEC for parents week 1 |  |  |  |
| 1. IEC for parents week 2 |  |  |  |
| 1. IEC for parents week 3 |  |  |  |
| 1. IEC for parents week 4 |  |  |  |
| 1. IEC for parents week 5 |  |  |  |
| 1. IEC for parents week 6 |  |  |  |
| 1. IEC for parents week 7 |  |  |  |
| 1. IEC for parents week 8 |  |  |  |
| 1. Activity for child week 1 |  |  |  |
| 1. Activity for child week 2 |  |  |  |
| 1. Activity for child week 3 |  |  |  |
| 1. Activity for child week 4 |  |  |  |
| 1. Activity for child week 5 |  |  |  |
| 1. Activity for child week 6 |  |  |  |
| 1. Activity for child week 7 |  |  |  |
| 1. Activity for child week 8 |  |  |  |
| *Mean score* | | *Mean score* | *Mean score* |
| *Range (0-32)* | | *Range (0-6)* | *Range (0-10)* |

**Supplementary Table 4. Standard Protocol Items: Recommendations for Interventional Trials (SPIRIT)**

|  | STUDY PERIOD (8 months) | | | | | | |
| --- | --- | --- | --- | --- | --- | --- | --- |
|  | *Enrolment* | *Allocation (2 months)* | | *Post-allocation* | | *Close-out (6 months)* | |
| TIMEPOINT | Baselines assessment |  |  | Immediately post- intervention assessment (t_0_) | | Endline Assessment (t_1_) | |
| *Study Groups* |  | *Intervention* | *Control* | *Intervention* | *Control* | *Intervention* | *Control* |
| *ENROLMENT:* |  |  |  |  |  |  |  |
| Eligibility screening | X |  |  |  |  |  |  |
| Informed consent | X |  |  |  |  |  |  |
| Allocation |  | X | X |  |  |  |  |
| *ASSESSMENTS:* |  |  |  |  |  |  |  |
| Screen-time |  |  |  | X | X | X | X |
| Sleep disturbances |  |  |  | X | X | X | X |
| Emotional behaviour |  |  |  | X | X | X | X |
| Physical activity |  |  |  | X | X | X | X |
| *INTERVENTIONS* |  |  |  | X | X | X | X |
| *Caregiver’s module* |  |  |  |  |  |  |  |
| 1. Information, education and communication material |  |  |  | X |  |  |  |
| 1. Counselling on digital screen exposure |  |  |  | X |  |  |  |
| 1. Digital media gadget placement at home |  |  |  | X |  |  |  |
| 1. Encouraging children on goal achievement |  |  |  | X |  |  |  |
| 1. Encouraging media free family-time and role-modelling by parents |  |  |  | X |  |  |  |
| 1. Suggesting alternatives to reducing digital media screens |  |  |  | X |  |  |  |
| 1. Positive reinforcement and rewarding children |  |  |  | X |  |  |  |
| *Child Module* |  |  |  |  |  |  |  |
| 1. Simple age-appropriate Mathematics questions |  |  |  | X |  |  |  |
| 1. Age-appropriate arts and craft activities |  |  |  | X |  |  |  |
| 1. Age-appropriate activities for learning language |  |  |  | X |  |  |  |
| 1. Introducing music and movement |  |  |  | X |  |  |  |
| 1. Goal-setting on reducing media-screens |  |  |  | X |  |  |  |
