# Supplementary material for: A Multicomponent Intervention to Reduce Screen Time Among Children Aged 2-5 Years in Chandigarh, North India: Protocol for a Randomized Controlled Trial
Source: JMIR Res Protoc. 2021 Feb 11;10(2):e24106. doi: 10.2196/24106 (PMC7906833; doi:10.2196/24106)
Supplement: Multimedia Appendix 7 [file resprot_v10i2e24106_app7.docx]

| Department of Community Medicine and School of Public Health,Post Graduate Institute of Medical Education & Research, PGIMER, Chandigarh, 160012 स्नातकोत्तरwebsite: <http://pgimer.nic.in/> | 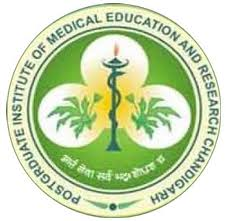 |
| --- | --- |

***Transtheoretical Model Proforma***

| 1. Do you think that your child is watching too much digital media? क्या आपको लगता है कि आपका बच्चा डिजिटल मीडिया उपकरणों का उपयोग बहुत ज्यादा कर रहा है | 1.1. Yes हाँ  1.2. No नहीं  1.3. May be शायद |
| --- | --- |
| 1. Have you ever thought that watching excessive digital media can affect the mental/ physical health of your child? क्या आपने कभी सोचा है कि अत्यधिक डिजिटल मीडिया देखने से आपके बच्चे के मानसिक/शारीरिक स्वास्थ्य पर असर पड़ सकता है? | 1.1. Yes हाँ  1.2. No नहीं  1.3. May be शायद |
| 1. Did you ever think of limiting the digital screen time of your child? क्या आपने कभी अपने बच्चे के डिजिटल स्क्रीन समय को सीमित करने के बारे में सोचा? |  |
| 1. Yes I am providing alternative to limit the excessive digital screen time since 1.1 हाँ मैं पिछले कुछ समय से अपने बच्चे को इन उपकरणों के इलावा और कुछ खेलने का सुझाव दे रहा हूँ | Week(s) सप्ताह / month(s) महीने |
| 1. Yes, I have planned to limit it but am not actively doing it हाँ, मैंने पिछले कुछ समय इसे सीमित करने की योजना बना रहा/रही हूँ लेकिन मैं इसे सक्रिय रूप से नहीं कर रहा हूं | Week(s) सप्ताह / month(s) महीने |
| 1. No, I have never thought about it नहीं, मैंने इसके बारे में कभी नहीं सोचा | Week(s) सप्ताह / month(s) महीने |
| 1. If no, then tell us the reason why you were not able to limit the child’s digital screen time? अगर नहीं, तो आप अपने बच्चे का इन उपकरणों पे बिताया गया समय क्यों नहीं कम कर पाए? |  |
| 1. If you have planned it, then tell us how do you plan to limit the excessive digital media? अगर अपने अपने बच्चे का इन उपकरणों पर बिताया गया समय कम कर लिया है, तो कृपया बताएँ कैसे? |  |
| 1. Reading books/ stories to the child बच्चे को किताब / कहानियाँ पढ़कर सुनाकर | 5.1.1 Yes हाँ 5.1.2 No नहीं |
| 1. Kept the child busy in playing inside the house such as colouring/ drawing/ painting etc. बच्चे को घर के अंदर खेलने में व्यस्त रख कर जैसे कि रंगना/ ड्राइंग / पेंटिंग आदि। | 5.2.1 Yes हाँ 5.2.2 No नहीं |
| 1. Promoting outside play such as sports/ active play etc. घर के बाहर जा कर खेल कूद के लिए प्रोत्साहित किया | 5.3.1 Yes हाँ 5.3.2 No नहीं |
| 1. Promoting socialization with others such as, play-dates, social gatherings etc. लोगों के साथ बातचीत करने के लिए प्रोत्साहित किया | 5.4.1 Yes हाँ 5.4.2 No नहीं |
| 1. Changes in the home environment such as, access to gadgets, placement of gadgets, control device etc घर में उपकरणों की जगह, रिमोट को अपने पास रखना, मोबाइल लॉक रखना इत्यादि में बदलाव किया | 5.5.1 Yes हाँ 5.5.2 No नहीं |
| 1. Restricting the digital-media duration and habits of the child at home. घर पर बच्चे की डिजिटल-मीडिया समय और आदतों पर रोक। | 5.6.1 Yes हाँ 5.6.2 No नहीं |
| 1. Others, specify अन्य, स्पष्ट करें | 5.7.1 Yes हाँ 5.7.2 No नहीं |
| Q6. If you are already doing the activities then please tell us what all alternatives are you providing the child to reduce the screen time among them along with the duration. यदि आप ये सब पहले से कर रहे हैं तो कब से कर रहे हैं कृपया स्पष्ट करें |  |
| - 1. Reading books/ stories to the child since बच्चे को किताब / कहानियाँ पढ़कर सुनाकर | Week(s) सप्ताह / month(s) महीने |
| - 1. Promoting inside play such as, coloring/ drawing/ painting etc. since बच्चे को घर के अंदर खेलने में व्यस्त रख कर जैसे कि रंगना/ ड्राइंग / पेंटिंग आदि। | Week(s) सप्ताह / month(s) महीने |
| - 1. Promoting outside play such as, sports/ active play etc. घर के बाहर जा कर खेल कूद के लिए प्रोत्साहित किया | Week(s) सप्ताह / month(s) महीने |
| - 1. Socializing with others such as, play-dates, social gatherings etc. since लोगों के साथ बातचीत करने के लिए प्रोत्साहित किया | Week(s) सप्ताह / month(s) महीने |
| - 1. Changes in the home environment such as, access to gadgets, placement of gadgets, control device etc. since घर में उपकरणों की जगह, रिमोट को अपने पास रखना, मोबाइल लॉक रखना इत्यादि में बदलाव किया | Week(s) सप्ताह / month(s) महीने |
| - 1. Restricting the digital-media duration and habits of the child at home. since घर पर बच्चे की डिजिटल-मीडिया समय और आदतों पर रोक। | Week(s) सप्ताह / month(s) महीने |
| - 1. Others, specify अन्य, स्पष्ट करें | Week(s) सप्ताह / month(s) महीने |
| Q7. For those who have been doing the activities for the last 6 months: Will you continue restrict the child in the same way in future also? in order to regulate your child’s screen time? उनके लिए जो इन सब दिए गए नियमों को पिछले 6 महीनों से रहे हैं: क्या आप आगे भी अपने घर में नियमों को लागू रखेंगे | 1.1. Yes हाँ  1.2. No नहीं  1.3. May be शायद |

**Interpretation**

| **Stage** | **Question and response** |
| --- | --- |
| **Precontemplation stage** | **Q1 =1.2; Q2=2.2; Q3=3.3** |
| **Contemplation stage** | **Q1= 1.1/1.3, Q2 = 2.1/2.3, Q3=3.2** |
| **Preparation stage** | **Q3=3.1, Q5=any (5.1/ 5.2/ 5.3/ 5.4/ 5.5/5.6/5.7)** |
| **Action stage** | **Q3=3.2, Q6= any [up to 6 months duration]** |
| **Maintenance stage** | **Q3=1, Q6= any [above 6 months]** |
| **Relapse** | **Q7= 7.2** |

Digital Screen Exposure Questionnaire (DSEQ)

**PART I. Personal Details (23 items) व्यक्तिगत जानकारी**

| 1. What is the name of the child? बच्चे का नाम क्या है? ________________________________________ | | | | | | |
| --- | --- | --- | --- | --- | --- | --- |
| 1. Who is the primary caregiver of the child? बच्चे की ज्यादातर समय देखभाल करने वाला कौन है?   1) Mother माँ 2) Father पिता 3) Grandfather दादा 4) Grandmother दादी 5) Other अन्य _______________ | | | | | | |
| 1. What is the marital status of the parents? माता-पिता की वैवाहिक स्थिति क्या है?   1) Married विवाहित 2) Widowed/ Widower विधवा/ विधुर 3) Divorced तलाकशुदा 4) Separated अलग  5) Never married कभी शादी नहीं की 6) Others please specify अन्य, कृपया उल्लेख/ वर्णन करें___________ | | | | | | |
| 1. What is the date of birth of the child: बच्चे की जन्मतिथि क्या है ____/____/________ **OR** या   Approximate age (in completed years) of the child बच्चे की आयु लगभग (पूरे किये साल में) कितने साल है ________ | | | | | | |
| 1. Gender of the child: बच्चे का लिंग क्या है: 1) Boy लड़का 2) Girl लड़की 3) Others अन्य | | | | | | |
| 1. Address पता: ____________________________________________________________________ | | | | | | |
| 1. What is your place of residence? आपका घर कहाँ है?   1) Urban शहर 2) Resettlement colony दुबारा बसी हुई कॉलोनी 3) Urbanised village छोटे शहर | | | | | | |
| 1. Total number of rooms in the house: घर में कमरों की कुल संख्या:___________________________ rooms | | | | | | |
| 1. Do you have a garden/ park area in/ near the house where the child can play?   क्या आपके घर के पास या घर में एक बगीचा/ पार्क है जहां बच्चा खेल सकता है? | | | | | Yes (हां)=1 | No (नही)=2 |
| 1. What is your Religion आपका धर्म क्या है? 1) Hindu हिंदू 2) Muslim मुस्लमान 3) Christian ईसाई 4) Sikh सिख 5) Others specify अन्य का उल्लेख/ वर्णन करें __________________________ | | | | | | |
| 1. What is your family type? आपका परिवार किस तरह का है 1) Nuclear family एकल परिवार 2) Joint family संयुक्त परिवार 3) Family with 3 generations (maternal/ paternal cousins) तीन पीढ़ियों वाला परिवार (ममेरे/ चचेरे भाई) | | | | | | |
|  | **13.1 Relation** रिश्ता | **12.2 Age** आयु | **12.3 *Education** शिक्षा | **12.4 ^#^Occupation** व्यवसाय | | **12.5 Income** आय |
| 12.01 | Father पिता |  |  |  | |  |
| 12.02 | Mother माता |  |  |  | |  |
| 12.03 | Head of the family (if other than the father)  परिवार का मुखिया (पिता नहीं है) |  |  |  | |  |
| 12.04 | Elder/ younger sister/ brother छोटे/ बड़े बहन/ भाई |  |  |  | |  |
| 12.05 | Elder/ younger sister/ brother छोटे/ बड़े बहन/ भाई |  |  |  | |  |
| 12.06 | Elder/ Younger sister/ brother छोटे/ बड़े बहन/ भाई |  |  |  | |  |
| **12.3 Education:** *1) Illiterate*: without any formal or non-formal education *2) Primary school literate*: those with non-formal education or those who joined school but did not study beyond 5^th^ standard *3) Middle school* *literate*: those who studies beyond 5^th^ standard but not beyond 8^th^ standard *4) High school literate*: those who have studied till 10^th^ standard *5) Intermediate Diploma*: those who studied till 10^th^ standard and completed a diploma course. Include those with technical education/ diplomas *6) Graduation*: those who have studiedtill graduation *7) Profession or Honors*: those who have studied beyond graduation.  12.3 शिक्षा: 1) अनपढ़: किसी भी औपचारिक या गैर-औपचारिक शिक्षा के बिना 2) प्राथमिक स्कूल साक्षर: गैर-औपचारिक शिक्षा वाले या जो स्कूल में शामिल हुए, लेकिन 5 वीं कक्षा से आगे की पढ़ाई नहीं की है 3) मिडिल स्कूल: वे जिन्होंने 5 वीं कक्षा से आगे की पढ़ाई की लेकिन 8 वीं कक्षा से आगे नहीं 4) हाई स्कूल: 10 वीं कक्षा तक की पढ़ाई करने वाले 5) इंटरमीडिएट डिप्लोमा: उन लोगों को जिन्होंने 10 वीं कक्षा तक पढ़ाई की और डिप्लोमा कोर्स पूरा किया। उन लोगों को शामिल करें जिन्होंने तकनीकी शिक्षा / डिप्लोमा पूरा किया 6) स्नातक: जिन लोगों ने स्नातक की पढ़ाई की है 7) व्यावसायिक या ऑनर्स की डिग्री: वे जो स्नातक से आगे की पढ़ाई कर चुके हैं।  **#12.4 Occupation:** 1) Legislators, senior officials and managers 2) Professionals 3) Technicians and associate professionals 4) Clerks 5) Skilled workers and shop/ market/ sales workers 6)Skilled agricultural & fishery workers 7) Craft and related trade workers: artisans, such as weavers, potters, painters, cobblers, shoe-makers, tailors, etc. 8) Plant & machine operators and assemblers: small scale or cottage industries, industrial/ factory worker, technician such as electricians, masons, plumbers, carpenters, goldsmiths, ironsmiths, those involved in automobile repair works etc., 9) Elementary occupation: includes workers on construction sites 10) Unemployed/ homemaker  # 12.4 पेशा: 1) विधायक, वरिष्ठ अधिकारी और प्रबंधक 2) पेशेवर 3) तकनीशियन और सहयोगी पेशेवर 4) क्लर्क 5) कुशल श्रमिक और दुकान / बाजार / बिक्री कार्यकर्ता 6) कुशल कृषि और मछली पालक 7) शिल्प और संबंधित व्यापार कार्यकर्ता: कारीगर, जैसे बुनकर, कुम्हार, चित्रकार, मोची, जूता बनाने वाले, दर्जी, आदि 8) प्लांट और मशीन चलाने वाले और बनाने/इकटठा करने वाले: छोटे पैमाने पर या कुटीर उद्योग, उद्योगिक / कारखाना कर्मचारी, तकनीशियन जैसे इलेक्ट्रीशियन, राजमिस्त्री, प्लंबर, बढ़ई, सुनार, लौहार, गाड़ियां की मरम्मत कार्यों में शामिल लोग आदि 9) प्राथमिक व्यवसाय: निर्माण स्थलों पर श्रमिक शामिल हैं 10) बेरोजगार / गृहिणी | | | | | | |
| 1. What is the socio-economic status of the family as per Kuppuswamy (refer to the guide)   परिवार की सामाजिक-आर्थिक स्थिति कुप्पुस्वामी (गाइड के संदर्भ में) के अनुसार क्या है ___________________ | | | | | | |
| 1. What is the total number of family members आपके परिवार के लोगों की कुल गिनती _________________ | | | | | | |
| Monthly family income **मासिक आय** 1) ≥78,063/- [12] **2)** 39,033-78062 [10] **3)** 29,200-39,032 [06]  **4)** 19,516-29,199 [04] **5)** 11,708-19,515 [02] **4)** ≤3907 [01] | | | | | | |
| 1. Per capita income of the family परिवार की प्रति व्यक्ति आय_____________________________ | | | | | | |
| 1. Child care facilities or services attended बाल देखभाल सुविधाएं | | | | | | |
| \|  \| 16.02 Number of days per week **उपस्थित दिनों की संख्या** हरेक सप्ताह \| 16.03 Duration/ day (total hours)  **कुल समय** हर **दिन (कुल घंटे)** \| \| --- \| --- \| --- \| \| 16.1 Informal child care (like, family members, nanny) आम बाल देखभाल (उदाहरण के लिए, परिवार के लोग, दाई ) \|  \|  \| \| 16.2 Formal care like नियमानुसार देखभाल जैसे कि (anganwadi आंगनवाड़ी, preschool शिशु स्कूल, play-way प्ले-वे etc.) \|  \|  \| \| 16.3 With the parents at home घर पर माता पिता के साथ \|  \|  \| \| 1. Do you own the following things? क्या आपके घर में यह चीज़ें हैं \| \| \| \|  \| 17.1 Yes हां=1 or या No नहीं=2 \| 17.2 Is the gadget usually placed in the room where the child sleeps/ plays? क्या गैजेट आमतौर पर उस कमरे में रखा जाता है जहां बच्चा घर पर सोता है / खेलता है? \| \| - 1. TV set with cable/ satellite connection टीवी केबल/ डिश कनेक्शन के साथ \|  \|  \| \| 17.02 Computer कम्प्यूटर/ Laptop लैपटॉप \|  \|  \| \| 17.03 Mobile phone without internet मोबाइल फोन \|  \|  \| \| 17.04 Smart phone with internet स्मार्ट फोन \|  \|  \| \| 17.05 Hand-held devices on which video-games can be played (tablet) हाथ में पकड़ने वाला उपकरण जिसपर वीडियो-गेम खेला जा सकता है (नोटबुक) \|  \|  \| \| 17.06 Internet connection (broadband/ WiFi) इंटरनेट कनेक्शन (ब्रॉडबैंड/ वाई-फाई) \|  \| **No answer expected here** \| | | | | | | |

| **PART II. SCREEN TIME EXPOSURE AND Home media environment (27 items) स्क्रीन पर बिताया समय और घर पर मीडिया का वातावरण** |
| --- |

| 1. Types of activities performed by the child, their duration and frequency   बच्चा क्या करता है, कब तक और कितनी बार करता है? | | | 18.1 Watches TV  टीवी देखना | 18.2 Uses a smart phone  स्मार्ट फोन का उपयोग | | 18.3 Uses other gadgets, specify  अन्य गैजेट्स का उपयोग, स्पष्ट करे | | 18.4 Writes/ draws/ colors  लिखना/ चित्र बनाना/ रंग भरना | | 18.5 Reads/ Read by someone  पढ़ना/ किसी द्वारा पढ़ाए जाना | | | 18.6 Others specify कोई भी अन्य | |
| --- | --- | --- | --- | --- | --- | --- | --- | --- | --- | --- | --- | --- | --- | --- |
| 18.01 Frequency of performing the activity in a given week; एक सप्ताह में गतिविधि कितनी बार करता है: 1) Never कभी नहीं 2) Rarely (less than once) शायद कभी (एक बार से भी कम) 3) seldom (1-2 times) शायद ही कभी (1-2 बार), 4) sometimes (3-4 times) कभी-कभी (3-4 बार), 5) Often (5 times or more) अक्सर (5 बार या अधिक | | |  |  | |  | |  | |  | | |  | |
| 18.02 Average duration on working/ school days per day (min) काम करने/ स्कूल वाले दिन प्रतिदिन में बिताया हुआ समय (मिनट में) | | |  |  | |  | |  | |  | | |  | |
| 18.03 Average duration on holidays per day (in min) छुट्टियों वाले दिन कितना समय (*मिनट में)* | | |  |  | |  | |  | |  | | |  | |
| 18.04 Whether the child does the above said activity supervised in a week? एक सप्ताह में बच्चा गतिविधि बड़े की देखरेख में करता है? 1) Never कभी नहीं 2) Rarely (less than once) शायद कभी (एक बार से भी कम) 3) seldom (1-2 times) शायद ही कभी (1-2 बार), 4) sometimes (3-4 times) कभी-कभी (3-4 बार), 5) Often (5 times or more) अक्सर (5 बार या अधिक) | | |  |  | |  | |  | |  | | |  | |
| 1. Which programs/ videos did the child watch yesterday बच्चे ने कल कौन से कार्यक्रम/वीडियो देखे? | | | A | | | B | | C | D | | | | | |
| 1. What was the duration of these programs/ videos इन कार्यक्रमों / वीडियो की अवधि क्या थी? | | |  | | |  | |  |  | | | | | |
| 1. Do you think the placement of TV in the room where the child plays/ sleeps can increases or decreases your child’s screen time? If no, skip to question 24   क्या आपको लगता है कि उस कमरे में, जहाँ बच्चा खेलता/ सोता है, टीवी रखने की जगह आपके बच्चे के स्क्रीन टाइम को बढ़ा या घटा सकता है? यदि नहीं तो प्रश्न संख्या 24 पर चले जाए | | | | | | | | | Yes हां=1 | | | No नही=2 | | |
| 1. If yes, then for how long is the TV switched on in the in the room where the child plays/ sleeps यदि हाँ, तो कितनी देर तक टीवी उस कमरे में चलता रहता है, जहाँ बच्चा खेलता/ सोता है। | | | | | | | | | | | | | | |
| 1. Do you have any rules regarding when, where, what & how to watch digital screen? (If no please skip to question 26)   क्या आपके पास कोई नियम हैं कि कब, कहाँ, क्या और कैसे डिजिटल स्क्रीन देखना है  *(यदि नहीं, तो कृपया प्रश्न 26 पर चले जाए)* | | | | | | | | | | | Yes हां=1 | No नही=2 | | |
| 1. If yes then, what rules do you have for the child at home? यदि हाँ तो घर में बच्चे की डिजिटल स्क्रीन देखने के लिए आपके पास क्या नियम हैं? | | | | | | | | | | |  |  | | |
| 24.1) Only children’s channel allowed केवल बच्चों के चैनल की अनुमति है | | | | | | | | | | |  |  | | |
| 24.2) The child isn’t allowed any media gadget 1h before sleep बच्चे को सोने से 1 घंटा पहले डिजिटल स्क्रीन देखने की अनुमति नहीं है | | | | | | | | | | |  |  | | |
| 24.3) The child is allowed only watch supervised by adults बच्चा केवल बड़ों की देखरेख में देखने की अनुमति है | | | | | | | | | | |  |  | | |
| 24.4) The child isn’t allowed to sit near the TV बच्चे को टीवी के नज़दीक बैठना मना है | | | | | | | | | | |  |  | | |
| 24.5) The child is allowed to watch only for a restricted duration बच्चे को निश्चित समय से अधिक देखने की अनुमति नहीं है __________________ min (*मिनट*) | | | | | | | | | | |  |  | | |
| 24.6) Any other reasons please specify अन्य कृपया उनका उल्लेख / वर्णन करें करे | | | | | | | | | | |  |  | | |
| 1. Caretaker’s media related factors देख-भाल करनेवाले की मीडिया संबंधी बातें | | | | | | | | | | | | | | |
| \|  \| Mother माता \| Father पिता \| \| --- \| --- \| --- \| \| 25.01 Average duration of screen time per day स्क्रीन समय की प्रतिदिन औसत अवधि \|  \|  \| \| 25.02 In a week what is the frequency of media gadget usage **एक सप्ताह में** डिजिटल मीडिया यंत्र उपयोग 1) Never **कभी नहीं** 2) Rarely (less than once) शायद कभी (एक बार से कम) 3) Seldom (1-2 times) **शायद ही कभी** (1-2 **बार**), 4) sometimes (3-4 times) **कभी-कभी** (3-4 **बार**) 5) Often (5 times or more) **अक्सर** (5 बार **या अधिक**) \|  \|  \| \| 25.03 Gadgets used 1) TV टीवी 2) Computer कम्प्यूटर 3) Laptop लैपटॉप 4) Tablet टेबलेट 6) Smart phone स्मार्ट फोन 7) Mobile phone मोबाइल फोन \|  \|  \| \| 25.04 Average time spent with the child at home घर पर बच्चे के साथ बिताया अंदाजन समय \|  \|  \| | | | | | | | | | | | | | | |
| PART III. PHYSICAL ACTIVITY RELATED QUESTIONS (10 items) गतिविधि संबंधित प्रश्न | | | | | | | | | | | | | | |
| 1. 01 Average duration of outside play per day on working/ school days (min) काम करने/ स्कूल के दिन कितना समय घर के भीतर खेलता (मिनट में) |  | 1. What was duration of the following outdoor activities that the child performed yesterday   निम्नलिखित बाहरी कामों की अवधि क्या थी जो बच्चे ने कल की थी | | | | | | | | | | | | |
| 26.02 Average duration on holidays of outside play per day (in min)  छुट्टियों वाले दिन कितना समय घर के भीतर खेलता (*मिनट में)* |  | - 1. Jogged quickly   हलकी सी तेजी से चलना | | | Yes हां=1 | No नही=2 | 27.05 Hopped easy  हलकी से कूदना | | | | | Yes हां=1 | | No नही=2 |
| 26.03 Total duration कुल समय |  | - 1. Tumbled moderately   धीरे से गिरना | | |  |  | 27.06 Hopped moderately  तेजी से कूदना | | | | |  | |  |
|  |  | - 1. Danced नाचना | | |  |  | 27.07 Hopped hard बड़ी तेजी से कूदना | | | | |  | |  |
|  |  | 27.04 Climbed चढ़ना | | |  |  | 27.08 Others अन्य | | | | |  | |  |

**PART IV. MEDIA RELATED BEHAVIORS** मीडिया के प्रति व्यवहार **(15 items)**

| \| 1. In a week, how frequently does the child watch this content on media gadgets at home?   एक सप्ताह में **घर पर मीडिया गैजेट्स पर बच्चा इस सामग्री को कितनी बार देखता है?** \| Never  कभी नहीं=1 \| Rarely (less than once) शायद कभी (एक बार से भी कम)=2 \| Seldom (1-2 times) शायद ही कभी (1-2 बार)=3 \| Sometimes (3-4 times) कभी-कभी (3-4 बार)=4 \| Often (5 times or more) अक्सर  **(5** बार या अधिक**)**=5 \| \| --- \| --- \| --- \| --- \| --- \| --- \| \| 1. The child uses for completing their homework assignments बच्चा कितनी बार स्कूल का काम करने क लिए मीडिया उपकरणों का इस्तेमाल करता है? \|  \|  \|  \|  \|  \| \| 1. The child uses video calling applications to talk to the family/ friends (skype, whatsapp etc.) बच्चा परिवार/ दोस्तों से वीडियो कॉल करने के लिए एप्लीकेशन (स्काइप, व्हाट्सएप, आदि) उपयोग करता है \|  \|  \|  \|  \|  \| \| 1. The child uses for learning poems, rhymes, ABC etc. online बच्चा इन उपकरणों का इस्तेमाल कवितायेँ, अक्षर सीखने के लिए करता है \|  \|  \|  \|  \|  \| \| 1. The child uses to learns maths, numbers, tables online. बच्चा इन उपकरणों का इस्तेमाल गणित, अंक, पहाड़े सीखने के लिए करता है \|  \|  \|  \|  \|  \| \| 1. The child uses to recognize shapes/ sounds/ colors when shown online बच्चा ऑनलाइन दिखाए जाने पर आकार/ आवाज़ / रंगों को पहचानने के लिए करता है \|  \|  \|  \|  \|  \| \| 1. The child learns various sciences online बच्चा विभिन्न विज्ञान से सम्बंधित चीजों को ऑनलाइन सीखने के लिए करता है \|  \|  \|  \|  \|  \| \|  \| Never  कभी नहीं=1 \| Rarely (less than once) शायद कभी (एक बार से भी कम)=2 \| Seldom (1-2 times) शायद ही कभी (1-2 बार)=3 \| Sometimes (3-4 times) कभी-कभी (3-4 बार)=4 \| Often (5 times or more) अक्सर  **(5** बार या अधिक**)**=5 \| \| 1. The child learns to draw, write online बच्चा चित्र बनाना, ऑनलाइन लिखना सीखने के लिए करता है? \|  \|  \|  \|  \|  \| \| 1. The child plays video-games बच्चा **वीडियो-गेम खेल**ता है? \|  \|  \|  \|  \|  \| \| 1. The child uses digital media gadgets to watch stories बच्चा इन उपकरणों का इस्तेमाल कहानियां देखने के लिए करता है \|  \|  \|  \|  \|  \| \| 1. The child to watch adult programs (soap opera, news, sports, movies etc.) बच्चा इन उपकरणों का इस्तेमाल वयस्क कार्यक्रम (ओपेरा,समाचार, खेल आदि) देखने के लिए करता है \|  \|  \|  \|  \|  \| \| 1. The child uses to learns letters, words, vocabulary, language online बच्चा अक्षर, शब्द, शब्दावली, भाषा सीखने के लिए करता है \|  \|  \|  \|  \|  \| \| 1. The child uses to watch random things for enjoyment (music, advertisements, babyTV, click photos etc.) बच्चा मनोरंजन के लिए बिना सोचे समझे देखने के लिए (संगीत, विज्ञापन, बच्चों का टीवी, फोटो खींचने आदि) करता है \|  \|  \|  \|  \|  \| \| 29. Which of the following does your child do as he/ she watches TV? नीचे लिखे में से कौन सा काम आपका बच्चा करता है जब वह टीवी देखता है? \| \| \| \| \| \| \| 29.1 a Talks about the program/ film कार्यक्रम/ फिल्म के बारे में बात करता है \|  \|  \|  \|  \|  \| \| 29.1 b Talks about other things अन्य चीजों के बारे में बात करता है \|  \|  \|  \|  \|  \| \| 29.1 c Talks to the character on the screen स्क्रीन पर आ रहे व्यक्ति/कलाकार से बात करता है \|  \|  \|  \|  \|  \| \| 29.2 Acts out the story/ role-play a character कहानी जैसे काम करता है/ कलाकार के जैसा व्यवहार करता है \|  \|  \|  \|  \|  \| \| 29.3 Sings गाता है \|  \|  \|  \|  \|  \| |
| --- | --- | --- | --- | --- | --- | --- | --- | --- | --- | --- | --- | --- | --- | --- | --- | --- | --- | --- | --- | --- | --- | --- | --- | --- | --- | --- | --- | --- | --- | --- | --- | --- | --- | --- | --- | --- | --- | --- | --- | --- | --- | --- | --- | --- | --- | --- | --- | --- | --- | --- | --- | --- | --- | --- | --- | --- | --- | --- | --- | --- | --- | --- | --- | --- | --- | --- | --- | --- | --- | --- | --- | --- | --- | --- | --- | --- | --- | --- | --- | --- | --- | --- | --- | --- | --- | --- | --- | --- | --- | --- | --- | --- | --- | --- | --- | --- | --- | --- | --- | --- | --- | --- | --- | --- | --- | --- | --- | --- | --- | --- | --- | --- | --- | --- | --- | --- | --- | --- | --- | --- |

**PART V. Media literacy of the parents (11 items)**

**माता-पिता की मीडिया के बारे में जानकारी**

| 1. What do you think are the good things that the child learns from these digital screens?   इन डिजिटल स्क्रीन को देखते समय माता-पिता को क्या लगता है कि बच्चा कौन सी अच्छी चीजों सीखता है? | | |
| --- | --- | --- |
|  | Yes हां=1 | No नही=2 |
| 30.1 The child is learning good habits बच्चा अच्छी आदतें सीख रहा है |  |  |
| 30.2 The child is Increasing his/ her knowledge बच्चा अपना ज्ञान बढ़ा रहा है |  |  |
| 30.3 The child is learning new skills बच्चा नई कला सीखता है |  |  |
| 30.4 It’s good for my child’s growth & development  यह बच्चे की तरक्की और विकास के लिए अच्छा है |  |  |
| 30.5 No positive effects कोई फायदा नहीं |  |  |
| 30.6 Any others specify कोई भी अन्य दिया गया कारण_____________________ |  |  |
| 1. What do you think are the problems when watching these screens excessively?   आपको क्या लगता है कि अधिक समय तक इन उपकरणों को देखने से बच्चे पर इसका बुरा असर हो सकता है? | | |
|  | Yes हां=1 | No नही=2 |
| 31.1 The child starts imitating what he watches  बच्चा जो देखता है उसकी नकल करना शुरू कर देता है |  |  |
| 31.2 The child develops sleep problems  बच्चे को नींद की समस्या हो सकती है |  |  |
| 31.3 The child might start eating unhealthy food  बच्चा अस्वास्थ्यकर भोजन खाना शुरू कर सकता है |  |  |
| 31.4 The child might become aggressive  बच्चा गुस्से वाला बन सकता है |  |  |
| 31.5 The child isolates himself/ herself  बच्चा आस पास की चीजों से अलग खुद को अलग कर लेता है |  |  |
| 31.6 It might Impairs the child’s concentration  यह बच्चे के किसी भी चीज पर ध्यान को कम कर सकता है |  |  |
| 31.7 It might cause behavior problems  यह व्यवहार की समस्याओं का कारण हो सकता है |  |  |
| 31.8 It might impair the child’s eyesight  बच्चे की नज़र को कमज़ोर कर सकता है |  |  |
| 31.9 It’s not good for my child’s growth/development  यह बच्चे की तरक्की/ विकास के लिए अच्छा नहीं है |  |  |
| 31.10 No negative effects कोई नुकसान नहीं |  |  |
| 31.11 Any others specify कोई अन्य का उल्लेख/ वर्णन करें _________________ |  |  |

**SLEEP DISTURBANCES SCALE FOR CHILDREN बच्चों की नींद संबंधी परेशानियों का विवरण**

***INSTRUCTIONS****: This questionnaire will allow to your doctor to have a better understanding of the sleep-wake rhythm of your child and of any problems in his/her sleep behavior. Try to answer every question; in answering, consider each question as pertaining to the* ***past 6 months*** *of the child’s life. Please answer the questions by circling or striking the number 1 to 5.*

निर्देश:यह प्रश्नावली आपके डॉक्टर को आपके बच्चे की नींद के बारे में किसी भी तरह की समस्या होने के बारे में जानकारी प्रदान करेगी। हर सवाल का जवाब देने की कोशिश करें; जवाब देने में, बच्चे के जीवन के पिछले 6 महीनों से संबंधित प्रत्येक प्रश्न पर विचार करें। कृपया 1 से 5 तक गोला लगाकर या ‘x’ का निशान लगा कर प्रश्नों का उत्तर दें।

| 1. How many hours of sleep does your child get on most nights. आपके बच्चे को ज्यादातर रातों में कितने घंटे की नींद आती है। | 1  *9-11 hours* घंटे | 2  *8-9 hours* घंटे | 3  *7-8 hours* घंटे | 4  *5-7 hours* घंटे | 5  *less than 5 hours* |
| --- | --- | --- | --- | --- | --- |
| 1. How long after going to bed does your child usually fall asleep आपका बच्चा आमतौर पर बिस्तर पर लेटने के कितने समय बाद सो जाता है | 1  *less than15' Min मिनट* | 2  *15-30' Min मिनट* | 3  *30-45' Min मिनट* | 4  *45-60' Min मिनट* | 5  *more than 60' मिनटों से ज्यादा* |

| 5 Always (daily) हमेशा (दैनिक) | | | | | |
| --- | --- | --- | --- | --- | --- |
| 4 Often (3 or 5 times per week) अक्सर (प्रति सप्ताह 3 या 5 बार) | | | | |  |
| 3 Sometimes (once or twice per week) कभी-कभी (प्रति सप्ताह एक या दो बार) | | | |  |  |
| 2 Occasionally (once or twice per month or less**)** कभी-कभी (एक या दो बार प्रति माह या उससे कम) | | |  |  |  |
| 1 Never कभी नहीं | |  |  |  |  |
| 1. The child goes to bed reluctantly   रात के सोने का समय होने पर भी बच्चा बिस्तर पर मुश्किल से जाता है | 1 | 2 | 3 | 4 | 5 |
| 1. The child has difficulty getting to sleep at night   बच्चे को रात को सोने में दिक्कत आती है | 1 | 2 | 3 | 4 | 5 |
| 1. The child feels anxious or afraid when falling asleep   सोते समय बच्चा घबराहट या डर महसूस करता है | 1 | 2 | 3 | 4 | 5 |
| 1. The child startles or jerks parts of the body while falling asleep   सोने की कोशिश करते समय बच्चे के शरीर को झटके लगते है या वो अचानक से डर के उठ जाता है | 1 | 2 | 3 | 4 | 5 |
| 1. The child shows repetitive actions such as rocking or head banging while falling asleep बच्चा सोते समय बार बार सिर हिलाता है या सिर पटकता है | 1 | 2 | 3 | 4 | 5 |
| 1. The child experiences vivid dream-like scenes while falling asleep   सोने की कोशिश करते हुए बच्चे को सपने आते है | 1 | 2 | 3 | 4 | 5 |
| 1. The child sweats excessively while falling asleep   सोने की कोशिश करते हुए बच्चे को बहुत ज्यादा पसीना आता है | 1 | 2 | 3 | 4 | 5 |
| 1. The child wakes up more than twice per night   बच्चा अधिकतर रातों को दो बार से ज्यादा **जाग** जा**ता** है | 1 | 2 | 3 | 4 | 5 |
| 1. After waking up in the night, the child has difficulty to fall asleep again   रात में जागने के बाद, बच्चे को फिर से सोने में कठिनाई होती है | 1 | 2 | 3 | 4 | 5 |
| 1. The child has frequent twitching or jerking of legs while asleep or often changes position during the night or kicks the covers off the bed.   सोते समय बच्चा पैरों को बार-बार हिलाता या मरोड़ता है या अक्सर रात को करवटें बदलता है या बिस्तर से चादर या तकिये भी हटा देता है। | 1 | 2 | 3 | 4 | 5 |
| 1. The child has difficulty in breathing during the night   बच्चे को रात के समय सांस लेने में कठिनाई होती है | 1 | 2 | 3 | 4 | 5 |
| 1. The child gasps for breath or is unable to breathe during sleep   बच्चा सांस लेने के लिए हांफता है या नींद के दौरान सांस नहीं ले पाता है | 1 | 2 | 3 | 4 | 5 |
| 1. The child snores   बच्चा खर्राटे लेता है | 1 | 2 | 3 | 4 | 5 |
| 1. The child sweats excessively during the night   बच्चे को रात के समय बहुत पसीना आता है | 1 | 2 | 3 | 4 | 5 |
| 1. You have observed the child sleepwalking   आपने बच्चे को नींद में चलते हुए देखा है | 1 | 2 | 3 | 4 | 5 |
| 1. You have observed the child talking in his/her sleep   आपने बच्चे को नींद में बातें करते हुए देखा है | 1 | 2 | 3 | 4 | 5 |
| 1. The child grinds teeth during sleep   बच्चा नींद के दौरान दांत पीसता है | 1 | 2 | 3 | 4 | 5 |
| 1. The child wakes from sleep screaming or confused so that you cannot seem to get through to him/her, but has no memory of these events the next morning   बच्चा रात को उठ कर चिल्लाना शुरू कर देता है, घबरा जाता है या उसे पता ही नहीं चलता कि क्या हो रहा है और आप उससे बात करना भी चाहो तो कर नहीं पाते और सुबह तक उसे कुछ भी याद नहीं रहता | 1 | 2 | 3 | 4 | 5 |
| 1. The child has nightmares which he/she doesn’t remember the next day   बच्चे को बुरे सपने आते हैं जो उसे अगले दिन याद नहीं रहते हैं | 1 | 2 | 3 | 4 | 5 |
| 1. The child is unusually difficult to wake up in the morning   बच्चे को सुबह उठाना जरुरत से ज्यादा मुश्किल होता है | 1 | 2 | 3 | 4 | 5 |
| 1. The child awakes in the morning feeling tired   बच्चा सुबह उठ के थका हुआ महसूस करता है | 1 | 2 | 3 | 4 | 5 |
| 1. The child feels unable to move when waking up in the morning   सुबह उठने पर बच्चा चलने-फिरने में दिक्कत महसूस करता है | 1 | 2 | 3 | 4 | 5 |
| 1. The child experiences daytime somnolence   बच्चे को दिन में नींद आती है | 1 | 2 | 3 | 4 | 5 |
| 1. The child falls asleep suddenly in inappropriate situations   बच्चा ऐसी जगहों पर अचानक सो जाता है जहाँ उसे सोना नहीं चाहिए | 1 | 2 | 3 | 4 | 5 |
|  | | | | | |
| Disorders of initiating and maintaining sleep (sum the score of the items 1,2,3,4,5,10,11) नींद शुरू करने और बनाए रखने के गड़बड़ी (वस्तुओं के अंकों का योग 1,2,3,4,5,10,11) |  | | | | |
| Sleep Breathing Disorders (sum the score of the items 13,14,15) नींद के दौरान सांस लेने में गड़बड़ी (वस्तुओं के अंकों का योग 13,14,15) |  | | | | |
| Disorders of arousal (sum the score of the items 17,20,21) उत्तेजना से गड़बड़ी (वस्तुओं के अंकों का योग 17,20,21) |  | | | | |
| Sleep-Wake Transition Disorders (sum the score of the items 6,7,8,12,18,19) नींद जाग संक्रमण गड़बड़ी (वस्तुओं के अंकों का योग 6,7,8,12,18,19) |  | | | | |
| Disorders of excessive somnolence (sum the score of the items 22,23,24,25,26) ज्यादा उदासीनता से गड़बड़ी (वस्तुओं के अंकों का योग 22,23,24,25,26) |  | | | | |
| Sleep Hyperhydrosis (sum the score of the items 9,16) नींद में अत्याधिक पसीना (वस्तुओं के अंकों का योग 9,16) |  | | | | |
| Total score (sum 6 factors’ scores) कुल अंकों (6 कारकों का योग) |  | | | | |

Please print. CHILD BEHAVIOR CHECKLIST FOR AGES 1 ½ -5 **कृपया छापें। बच्चे का व्यवहार जांच-सूची 1½-5 उम्र के बच्चों के लिए** For office use only केवल कार्यालय उपयोग के लिए

| Below is a list of items that describe children. For each item that describes the child now or within the past 2 months, please circle the 2 if the item is very true or often true of the child. Circle the 1 if the item is somewhat or sometimes true of the child. If the item is not true of the child, circle the 0. Please answer all items as well as you can, even if some do not seem to apply to the child.  नीचे दी गई चीजों की सूची बच्चों के बारे में वर्णन करती हैं। प्रत्येक चीज जो बच्चे का अभी या पिछले 2 महीनों के भीतर का वर्णन करती है, कृपया 2 पर गोला लगाए यदि वो चीज अक्सर ही बच्चे के बारे में सच है। यदि वो चीज बच्चे के बारे में कुछ हद तक या कभी-कभी सच है, तो 1 पर गोला लगाए। यदि वो चीज बच्चे के लिए सही नहीं है, तो 0 पर गोला लगाए। कृपया सभी चीजों के उत्तर दें, भले ही यह बच्चे पर लागू न हों।  **0 = Not True (as far as you know) सच नहीं (जहाँ तक आप जानते हैं) 1 = Somewhat or Sometimes True कुछ हद तक या कभी-कभी सच 2=Very True or Often True बहुत सच या अक्सर सच** |
| --- |

| 0 | 1 | 2 | 1. | Aches or pains (without medical cause; **do not** include stomach or headaches)  दर्द महसूस करना (बिना चिकित्सीय कारण; पेट या सिर दर्द को शामिल मत करें) |
| --- | --- | --- | --- | --- |
| 0 | 1 | 2 | 2. | Acts too young for age उम्र के हिसाब से अपने से कम उम्र के बच्चों की तरह व्यवहार करता/करती है |
| 0 | 1 | 2 | 3. | Afraid to try new things नई चीजों को करने में डरता/डरती है |
| 0 | 1 | 2 | 4. | Avoids looking others in the eye दूसरों से आँखें मिलाने से बचता/बचती है |
| 0 | 1 | 2 | 5. | Can’t concentrate, can’t pay attention for long किसी भी एक चीज पर ध्यान केंद्रित नहीं कर पाता/पाती, लंबे समय तक ध्यान नहीं दे पाता/पाती |
| 0 | 1 | 2 | 6. | Can’t sit still, restless, or hyperactive लंबे समय के लिए नहीं बैठ सकता/सकती, बेचैन सा रहता/रहती है या जरुरत से ज्यादा हिलता डुलता/हिलती डुलती है |
| 0 | 1 | 2 | 7. | Can’t stand having things out of place अस्त व्यस्त चीजों को बर्दाश्त नहीं कर सकता/सकती |
| 0 | 1 | 2 | 8. | Can’t stand waiting;wants everything now इंतजार नहीं कर सकता/सकती; सब कुछ उसी समय चाहता/चाहती है |
| 0 | 1 | 2 | 9. | Chews on things that aren't edible उन चीजों को भी चबाता/चबाती है जो खाने योग्य नहीं हैं |
| 0 | 1 | 2 | 10. | Clings to adults or too dependent बड़ों पर निर्भर रहता/रहती है या उनके साथ चिपका रहता/रहती है |
| 0 | 1 | 2 | 11. | Constantly seeks help लगातार मदद मांगता/मांगती है |
| 0 | 1 | 2 | 12. | Constipated,doesn’t move bowels (when not sick) कब्ज़ रहती है, टट्टी नहीं आती (जब बीमार नहीं होता/होती) |
| 0 | 1 | 2 | 13. | Cries a lot खूब रोता/रोती है |
| 0 | 1 | 2 | 14. | Cruel to animals जानवरों के साथ क्रूरता के साथ पेश आता/आती है |
| 0 | 1 | 2 | 15. | Defiant विरोध करता/करती है |
| 0 | 1 | 2 | 16. | Demands must be met immediately उम्मीद करता/करती है कि मांगों को तुरंत माना जाना चाहिये |
| 0 | 1 | 2 | 17. | Destroys his/her own things खुद की चीजों को नुकसान पहुंचाता/पहुंचाती है |
| 0 | 1 | 2 | 18. | Destroys things belonging to his/her family or other children अपने परिवार या अन्य बच्चों की चीजों को नुकसान पहुंचाता/पहुंचाती है |
| 0 | 1 | 2 | 19. | Diarrhea or loose bowels (when not sick) दस्त लगते है या बार बार टट्टी जाता/जाती है (जब बीमार न हों) |
| 0 | 1 | 2 | 20. | Disobedient बात नहीं मानता/मानती |
| 0 | 1 | 2 | 21. | Disturbed by any change in routine दिनचर्या में किसी भी बदलाव से परेशान हो जाता/जाती है |
| 0 | 1 | 2 | 22. | Doesn’t want to sleep alone अकेले सोना नहीं चाहता/चाहती है |
| 0 | 1 | 2 | 23. | Doesn’t answer when people talk to him/her जब लोग उससे बात करते हैं तो जवाब नहीं देता/देती |
| 0 | 1 | 2 | 24. | Doesn’t eat well (describe): अच्छी तरह से खाना नहीं खाता/खाती (वर्णन): ______ ________________________________________________________________ |
| 0 | 1 | 2 | 25. | Doesn’t get along with other children दूसरे बच्चों के साथ घुलता-मिलता/घुलती मिलती नहीं |
| 0 | 1 | 2 | 26. | Doesn’t know how to have fun; acts like a little adult मस्ती कैसे करते है नहीं जानता/जानती, बड़ों की तरह व्यवहार करता/करती है |
| 0 | 1 | 2 | 27. | Doesn’t seem to feel guilty after misbehaving गलत व्यव्हार करने के बाद भी अपनी गलती पर शर्मिन्दा महसूस नहीं करता/करती |
| 0 | 1 | 2 | 28. | Doesn’t want to go out of home घर से बाहर नहीं जाना चाहता/चाहती |
| 0 | 1 | 2 | 29. | Easily frustrated जल्दी परेशान हो जाता/जाती है |
| 0 | 1 | 2 | 30. | Easily jealous आसानी से ईर्ष्या हो जाती है |
| 0 | 1 | 2 | 31. | Eats or drinks things that are not food-**don’t** include sweets (describe): ऐसी चीजें खाता/खाती या पीता/ पीती है जोकि खाने वाली नहीं होती। मिठाई शामिल न करें (वर्णन करें): ___________ |
| 0 | 1 | 2 | 32. | Fears certain animals, situations, or places(describe): कुछ जानवरों, स्थितियों या जगहों से डर लगता है (वर्णन करें): ______________________ |
| 0 | 1 | 2 | 33. | Feelings are easily hurt भावनाओं को जल्दी चोट लग जाती है |
| 0 | 1 | 2 | 34. | Gets hurt a lot, accident-prone बहुत चोटें लगती है |
| 0 | 1 | 2 | 35. | Gets in many fights अक्सर झगड़ों में पड जाता/जाती है |
| 0 | 1 | 2 | 36. | Gets into everything हर काम में शामिल हो जाता/जाती है |
| 0 | 1 | 2 | 37. | Gets too upset when separated from parents माता-पिता से अलग होने पर बहुत परेशान हो जाता/जाती है |
| 0 | 1 | 2 | 38. | Has trouble getting to sleep नींद में दिक्कत होती है |
| 0 | 1 | 2 | 39. | Headaches (without medical cause) सिरदर्द होता है (बिना चिकित्सकीय कारण के) |
| 0 | 1 | 2 | 40. | Hits others दूसरों को मारता/मारती है |
| 0 | 1 | 2 | 41. | Holds his/her breath अपनी साँसे रोक लेता/लेती है |
| 0 | 1 | 2 | 42. | Hurts animals or people without meaning toबिना मतलब के जानवरों या लोगों को चोट पहुँचाता/पहुँचाती है |
| 0 | 1 | 2 | 43. | Looks unhappy without good reason बिना कारण के ही दुखी नज़र आता/आती है |
| 0 | 1 | 2 | 44. | Angry moods गुस्से वाला स्वभाव |
| 0 | 1 | 2 | 45. | Nausea, feels sick (without medical cause) जी मिचलता है, अपने आप में बीमार महसूस करता/करती है (चिकित्सा कारणों के बिना) |
| 0 | 1 | 2 | 46. | Nervous movements or twitching (describe): घबराहट या झटके (वर्णन):___________ |
| 0 | 1 | 2 | 47. | Nervous, highstrung or tense बेचैन, भावुक और परेशान |
| 0 | 1 | 2 | 48. | Nightmares बुरे सपने आते हैं |
| 0 | 1 | 2 | 49. | Overeating ज्यादा खाता/खाती है |
| 0 | 1 | 2 | 50. | Overtired ज्यादा थका हुआ महसूस करता/करती है |
| 0 | 1 | 2 | 51. | Shows panic for no good reason छोटी सी बात पर भी बहुत घबराता/घबराती है |
| 0 | 1 | 2 | 52. | Painful bowel movements (without medical cause) टट्टी करते हुए दर्द बताता/ बताती है (चिकित्सीय कारण के बिना) |
| 0 | 1 | 2 | 53. | Physically attacks people दूसरों को मारता पीटता/मारती पीटती है |
| 0 | 1 | 2 | 54. | Picks nose, skin, or other parts of body (describe): नाक, त्वचा, या शरीर के अन्य हिस्सों को नोचता/नोचती रहता/ रहती है (वर्णन): ____________________ |
| 0 | 1 | 2 | 55. | Plays with own sex parts too much खुद के यौन अंगों के साथ बहुत ज्यादा खेलता/ खेलती है |
| 0 | 1 | 2 | 56. | Poorly coordinated or clumsy चीजों को करते वक़्त सही तरीके से नहीं कर पाता/ पाती। उसके हाथ पैर सही तरीके से नही चलते |

Be sure you answered all items. Then see other side. **सुनिश्चित करें कि आपने सभी आइटमों का दे उत्तर दिया है। फिर दूसरा तरफ देखें।**

| 0 | 1 | 2 | 57. | Problems with eyes (without medical cause) (describe): आँखों की समस्याएँ (बिना चिकित्सीय कारण) (वर्णन):_______________________________ |
| --- | --- | --- | --- | --- |
| 0 | 1 | 2 | 58. | Punishment doesn’t change his/her behavior सजा उसका व्यवहार नहीं बदलती है |
| 0 | 1 | 2 | 59. | Quickly shifts from one activity to another किसी भी एक काम को ज्यादा देर तक टिक कर नहीं करता/करती है और उसको छोड़कर दूसरी चीज करने लगता/लगती है |
| 0 | 1 | 2 | 60. | Rashes or other skin problems (without medical cause) चमड़ी की समस्या रहती है जैसे कि चमड़ी पर निशान पड़ना या दाने होना (बिना चिकित्सकीय कारण के) |
| 0 | 1 | 2 | 61. | Refuses to eat खाने के लिए मना कर देता/देती है |
| 0 | 1 | 2 | 62. | Refuses to play active games जिस खेल में बहुत भागना पड़ता है उसे खेलने के लिए मना कर देता/देती है |
| 0 | 1 | 2 | 63. | Repeatedly rocks head or body बार-बार सिर या शरीर पटकता/पटकती है |
| 0 | 1 | 2 | 64. | Resists going to bed at night रात में बिस्तरपर जाने से कतराता/कतराती है |
| 0 | 1 | 2 | 65. | Resists toilet training (describe): टट्टी पेशाब को नियमित समय पर करने का विरोध करता/करती है (वर्णन): _____________________________________________ |
| 0 | 1 | 2 | 66. | Screams a lot बहुत चिल्लाता/ चिल्लाती है |
| 0 | 1 | 2 | 67. | Seems unresponsive to affection प्यार या स्नेह का जवाब नहीं देता लगता/देती लगती है |
| 0 | 1 | 2 | 68. | Self-conscious or easily embarrassed परेशान या आसानी से शर्मिंदा महसूस करता/करती है |
| 0 | 1 | 2 | 69. | Selfish or won’t share स्वार्थी या साझा नहीं करता/करती |
| 0 | 1 | 2 | 70. | Shows little affection toward people लोगों के प्रति बहुत कम स्नेह दिखाता/दिखाती है |
| 0 | 1 | 2 | 71. | Shows little interest in things around him/her उसके आसपास की चीजों में बहुत कम रुचि दिखाता/दिखाती है |
| 0 | 1 | 2 | 72. | Shows too little fear of getting hurt चोट लगने का बहुत कम डर है |
| 0 | 1 | 2 | 73. | Too shy or timid बहुत शर्मीला/शर्मिली या डरने वाला/वाली |
| 0 | 1 | 2 | 74. | Sleeps less than most kids during day and/or night (describe): अधिकांश बच्चों की तुलना में उसे कम नींद आती है (वर्णन करें):_______________________ |
| 0 | 1 | 2 | 75. | Smears or plays with bowel movements टट्टी के साथ खेलता/खेलती है या गंदा करता/करती है |
| 0 | 1 | 2 | 76. | Speech problem (describe): बोलने की दिक्कत है (वर्णन): ____________________ |
| 0 | 1 | 2 | 77. | Stares into space or seems preoccupied आसमान को घूरता रहता/रहती है या बेचैन रहता/रहती है |
| 0 | 1 | 2 | 78. | Stomachaches or cramps (without medical cause) पेट में दर्द या मरोड़ (बिना चिकित्सकीय कारण के) |
| 0 | 1 | 2 | 79. | Rapid shifts between sadness and excitement उदासी और उत्तेजना के बीच तेजी से बदलाव |
| 0 | 1 | 2 | 80. | Strange behavior (describe): अजीब व्यवहार (वर्णन): _______________________ |
| 0 | 1 | 2 | 81. | Stubborn, sullen, or irritable जिद्दी, उदास या चिड़चिड़ा |
| 0 | 1 | 2 | 82. | Sudden changes in mood or feelings अचानक मूड या भावनाओं में बदलाव |
| 0 | 1 | 2 | 83. | Sulks a lot बहुत कुछ करता/करती है |
| 0 | 1 | 2 | 84. | Talks or cries out in sleep नींद में बात करता/करती है या रोता/रोती है |
| 0 | 1 | 2 | 85. | Temper tantrums or hot temper गुस्सा नखरे या गर्म स्वभाव |
| 0 | 1 | 2 | 86. | Too concerned with neatness or cleanliness बहुत साफ-सफाई या स्वत्छता पसंद करता/करती है |
| 0 | 1 | 2 | 87. | Too fearful or anxious बहुत भयभीत या चिंतित |
| 0 | 1 | 2 | 88. | Uncooperative दूसरों को सहयोग नहीं करता/करती है |
| 0 | 1 | 2 | 89. | Underactive, slow moving, or lacks energy चंचल नहीं है, धीमी गति से चलना या ऊर्जा की कमी |
| 0 | 1 | 2 | 90. | Unhappy, sad or depressed नाखुश, दुखी या उदास |
| 0 | 1 | 2 | 91. | Unusually loud आमतौर से ज्यादा जोर से बोलने वाला/वाली |
| 0 | 1 | 2 | 92. | Upset by new people or situations (describe): नए लोगों या स्थितियों से परेशान (वर्णन करें):______________________________________ |
| 0 | 1 | 2 | 93. | Vomiting, throwing up (without medical cause) उल्टी, खाई हुई चीजें निकाल देता/देती है (बिना चिकित्सकीय कारण के) |
| 0 | 1 | 2 | 94. | Wakes up often at night रात में अक्सर उठ जाता/जाती है |
| 0 | 1 | 2 | 95. | Wanders away भटक कर कहीं भी चला जाता/जाती है |
| 0 | 1 | 2 | 96. | Wants a lot of attention बहुत ध्यान चाहता/चाहती है |
| 0 | 1 | 2 | 97. | Whining हमेशा शिकायत करता/करती है |
| 0 | 1 | 2 | 98. | Withdrawn, doesn’t get involved with others दूर-दूर रहता/रहती है, दूसरों के साथ घुलता मिलता/मिलती नहीं |
| 0 | 1 | 2 | 99. | Worries बहुत सोचता/सोचती है |
| 0 | 1 | 2 | 100. | Please write in any problems the child has that were not listed above. कृपया बच्चे की उन समस्याऔ के बारे में लिखें जिसे ऊपर नहीं बताया गया है। |
| 0 | 1 | 2 |  | _______________________________ |
| 0 | 1 | 2 |  | _______________________________ |
| 0 | 1 | 2 |  | _______________________________ |

**Please be sure you have answered all items.** कृपया सुनिश्चित करें कि आपने सभी आइटमों का उत्तर दिया है।

Underline any you are concerned about. आप जिस किसी के बारे में चिंतित हैं उसे रेखांकित करें।

Does the child have any illness or disability (either physical or mental)? □ No □ Yes —Please describe: क्या बच्चे को कोई बीमारी या विकलांगता है (या तो शारीरिक या मानसिक)? □ नहीं □ हां-कृपया वर्णन करें:

__________________________________________________________________________________________

What concerns you most about the child? आपको बच्चे के बारे में सबसे ज्यादा क्या चिंता रहती है?

___________________________________________________________________________________________

Please describe the best things about the child: कृपया बच्चे के बारे में सबसे अच्छी बातों के बारे में बताएं:
