# Supplementary material for: A Multicomponent Intervention to Reduce Screen Time Among Children Aged 2-5 Years in Chandigarh, North India: Protocol for a Randomized Controlled Trial
Source: JMIR Res Protoc. 2021 Feb 11;10(2):e24106. doi: 10.2196/24106 (PMC7906833; doi:10.2196/24106)
Supplement: Multimedia Appendix 8 [file resprot_v10i2e24106_app8.pdf]

TRB. 886

22/2/17

52

School of Public Health
Post Graduate Institute of Medical Education & Research, Chandigarh
Minutes of the First Doctoral Committee Meeting

Name of the Candidate: Dr. NimranKaur
Date of Registration: 29th July 2016
Period of Registration: 3 years
Title of the plan of Thesis: **"Effectiveness of a Multi- Component Intervention to Reduce Screen-Based Sedentary Behaviors' in Children aged 2-5 years of Chandigarh, Union Territory: Cluster Randomized Control Trial".**

Doctoral Committee Members

1. Dr. A[illegible]Bhalla	Chairman
2. Dr. Manoj R Kumar	Member
3. Dr. Bhavneet Bharti	Member
4. Dr. Manmeet Kaur	Member
5. Dr. Arun Bansal	Member
6. Dr. Vikas Suri	Member
7. Dr. Shankar Prinja	Member
8. Dr. Madhu Gupta	Convener
9. Dr. Prahbhjot Malhi	Co- Guide
10. Dr. S[illegible]deep Grover	Co- Guide

Doctoral Committee Meeting was held on 13th January 2017 from 2.30 to 4.30 pm in Committee Room of Department of Community Medicine, School of Public Health PGIMER, Chandigarh. Dr. Anil Bhalla chaired the meeting. Dr. Madhu Gupta, Dr. Bhavneet Bharti, Dr. Manmeet Kaur, Dr. Arun Bansal, Dr. Shankar Prinja and special invitees Dr. Prahbhjot Malhi (co-guide), Dr. Sandeep Grover (co-guide) attended the meeting. Dr. Manoj R Kumar and Dr. Vikas Suri could not attend the meeting due to prior commitments.

En

The following modifications were suggested:

1. The re[illegible]iew of literature needs to be strengthened. Studies on factors associated with increased screen time, consequences of increased screen time on children's emotional and social behavior needs to be described in detail in the protocol.
2. It is suggested that there is no need to validatethe tool to measure the screen time among children of age 2-5 years, as the screen time questionnaire is not a scale. It is suggested to only pretest this questionnaire.
3. Hence, aims and objectives of the study need to be revised accordingly.

22/2/17

Scanned with CamScanner

4. Socioeconomic status related questions using modified Kuppuswamy scale needs to be added in the screen time questionnaire.
5. It is also advised to include the questions that can provide information on alternatives parents/caregivers would like to propose for reducing the screen time among children, especially by working parents/caregivers in the screen time questionnaire.
6. Inclusion/ exclusion criteria in both prevalence and intervention study need to be revised from 2 to 5 years to 2years ±3 months to 5 years± 3 months
7. Sample size for the prevalence study needs to be revised as per availability of prevalence studies in the local settings, if no local prevalence studies are available then 50% prevalence can be assumed for sample size calculations.
8. Population of Chandigarh as per Census 2011 needs to be given in the protocol. Same termin[illegible]logy is to be used for classifying rural, urban and slum areas of Chandigarh as used in the census 2011. The stratified sampling technique needs to be revised accordingly.
9. For calculating the sample size for intervention study, it was suggested to calculate intra cluster coefficient (ICC) after pilot testing the intervention in the local setting.
10. It was suggested to include the type of study design in the title.
11. Data analyses plan needs to be elaborated for prevalence and intervention study.

Dr. Anil Bhalla

Dr. A. K. Bhalla
Professor
Additional Medical Supdt.
APC PGIMER, Chandigarh.

प्राध्यापक
Professor
बाल रोग विज्ञान विभाग
Deptt. of Pediatrics
पी०जी०आई०,चण्डीगढ़-160 012
PGIMER, Chandigarh-160 012

Dr. Bhavneet Bharti

Dr. Manmeet Kaur
[illegible] PROFESSOR
DEPTT. OF COMMUNITY [illegible]
PGIMER, CHANDIGARH

[illegible]
Additional Professor
बाल रोग विज्ञान विभाग
Deptt. of Pediatrics
[illegible]
PGIMER, Chandigarh-160 012 (India)

Dr. Arun Bansal

Dr. Shankar Prinja
ASSOCIATE PROFESSOR
COMMUNITY MEDICINE DE[illegible]
PGIMER, CHANDIGARH

Dr. Sandeep Grover

Dr. Prahbhjot Malhi

डॉ. संदीप ग्रोवर
Dr. Sandeep Grover
अपर प्राचार्य
Addl. Professor
मनोचिकित्सा विभाग
Deptt. of Psychiatry
स्नातकोत्तर चिकित्सा शिक्षा एवं अनुसंधान संस्थान, चण्डीगढ़ ।
PGIMER, Chandigarh

Dr. Madhu Gupta
ADDITIONAL PROFESSOR
DEPTT. OF COMMUNITY MEDICINE
PGIMER, CHANDIGARH

प्राध्यापक
Professor
बाल रोग विज्ञान विभाग
Deptt. of Pediatrics
पी०जी०आई०,चण्डीगढ़-160 01[illegible]
PGIMER, Chandigarh-160 01[illegible]

Scanned with CamScanner

Responses to the comments of the First Doctoral Committee

Comment 1: The review of literature needs to be strengthened. Studies on factors associated with increased screen time, consequences of increased screen time on children's emotional and social behavior needs to be described in detail in the protocol.

Response 1: Studies on factors associated with increased screen time, consequences of increased screen time added in the review of literature section and described in details from Page 9 to 16.

Comment 2: It was suggested that there is no need to validate the tool to measure the screen time among children of age 2-5 years. It is suggested to only pretest the questionnaire.

Response 2: It has been revised accordingly.

Comment 3: Hence, aims and objectives of the study need to be revised accordingly.

Response 3: The aims and objectives are also revised accordingly on Page 27.

Comment 4: Socioeconomic status related questions using modified scale needs to be added in the questionnaire.

Response 4: It is added in the questionnaire as question number 17 in Annexure I and also mentioned in the protocol on Page 30.

Comment 5: It is also advised to include the questions that can provide information on alternatives parents/ caregivers' would like to propose for reducing the screen time among children, especially by working parents/ caregivers' in the screen time questionnaire.

Response 5: It is included in the questionnaire as question number 27 in Annexure I

Comment 6: Inclusion/ exclusion criteria in both prevalence and intervention study need to be revised so as to revise the age criteria from 2 to 5 years to 2 years ± 3 months to 5 years ± 3 months

Response 6: Inclusion/ exclusion criteria in both prevalence (given on Page 28 and 29) and intervention study (given on Page 33) revised from 2 to 5 years to 2 years ± 3 months to 5 years ± 3 months as suggested.

Comment 7: Sample size for the prevalence study needs to be revised as per availability of prevalence studies in the local settings, if no local prevalence studies are available then 50% prevalence can be assumed for sample size calculations.

Scanned with CamScanner

Response 7: As there are no local studies available on the prevalence of screen time, hence prevalence is assumed to be 50% for calculating the sample size of this prevalence study as given on Page 29.

Comment 8: Population of Chandigarh as per Census 2011 needs to be given in the protocol. Same terminology is to be used for classifying rural, urban and slum areas of Chandigarh as used in the Census 2011. The stratified sampling technique needs to be revised accordingly.

Response 8:

- Population of Chandigarh is revised as per Census 2011 on Page 28
- The terminology and sampling strategy is revised as per Census 2011 on Page 29 & 30

Comment 9: For calculating the sample size for the intervention study, it was suggested to calculate intracluster coefficient (ICC) after pilot testing the intervention in the local setting.

Response 9: Details regarding pilot testing of cluster randomized control trial (c-RCT) is now described on Page 34.

Comment 10: It was suggested to include the type of study design in the title.

Response 10: The study title is revised " A Cluster Randomized Control Trial to Reduce Screen-Based Sedentary Behaviors' in 2-5 years Children of Chandigarh" as suggested.

Comment 11: Data analyses plan needs to be elaborated for prevalence and intervention study.

Response 11: Detailed data analyses plan has been revised in the protocol for prevalence study on Page 31 and intervention study on Page 36.

Scanned with CamScanner

Minutes of the 2nd Doctoral Committee meeting

The 2nd Doctoral Committee meeting of Ms. Nimran Kaur, PhD student at Department of Community, Medicine, School of Public Health was held on **11th September 2018 at 3:00 pm** in Committee Room of Advanced Pediatric Centre (Room number- 3105), PGIMER, Chandigarh. The session was chaired by Dr. Anil Bhalla, Professor & Assistant Medical Superintendent, Advanced Pediatric Centre, PGIMER, Chandigarh. The following members is the list of committee members, and special invitees for the attend the meeting:

1.	Dr. Anil Bhalla	Chairman
2.	Dr. Bhavneet Bharti	Member
3.	Dr. Manmeet Kaur	Member
4.	Dr. Vikas Suri	Member
5.	Dr. Arun Bansal	Member
6.	Dr. Manoj K. Rohit	Member
7.	Dr. Shankar Prinja	Member
8.	Dr Madhu Gupta	Chief guide
9	Dr. Prahbhjot Malhi	Co-guide
10.	Dr. Sandeep Grover	Co-guide

Out of the total eight members, five members were present Dr. Anil Bhalla (Professor, Advanced Pediatric Centre), Dr. Manmeet Kaur (Professor, School of Public Health & Department of Community Medicine), Dr. Vikas Suri (Professor, Department of Internal Medicine), Dr. Arun Bansal (Professor, Advanced Pediatric Centre), Dr. Madhu Gupta (Professor, School of Public Health & Department of Community Medicine) and special invitee Dr. Sandeep Grover (Professor, Department of Psychiatry). The members suggested the following changes in the study:

1. It was suggested to carefully, and authentically estimate the time of screen exposure, and mention it in detail in the methods in the prevalence and intervention study.
2. It was suggested to revise the following points in the analysis of the prevalence study:
   - 2.1. To change the age ranges to below 3 years, 4 years, and 5 years as the definition of toddlers, and preschoolers is variable worldwide.
   - 2.2. To estimate the correlations of screen-time with sleep, emotional disturbances, and physical activity, as no specific guidelines for screen exposure exist in India.
   - 2.3. Mention specifically in the exclusion criteria that cinema/ movies were not included while estimating the time for digital media exposure.
3. To change the study design from cluster-Randomized control trial (c-RCT) to Randomized control trial (RCT) as the study sample (n 6800) would not be feasible with the estimated intra cluster

Scanned with CamScanner

coefficient of 0.3. Make the study representative of the whole population of Chandigarh, so that the results can be generalized.

4. If a randomized control trial (RCT) is planned in the field practice area of Department of Community Medicine and School of Public Health, then conduct a pilot study to verify the prevalence of digital screen exposure in that study area, so that its results can be compared with the main prevalence study results.
5. To incorporate focused group discussions (FGDs) with the parents to devise a better intervention plan in the designing phase.
6. To elaborate the strategies to motivate parents in proposing alternatives to their children in their respective homes in the methods of the intervention study.
7. It was suggested to compare the screen-related literacy of the parents at the start, and end of the intervention.
8. Lastly, the committee members appreciated the findings of the study and emphasized on publishing the results of the same in a reputed, and indexed journal.

Dr. Anil Bhalla

Dr. A. K. Bhalla
Professor
Additional Medical Supdt.
APC PGIMER, Chandigarh.

डॉ संदीप ग्रोवर
Dr. Sandeep Grover
प्राचार्य
Professor
[illegible] विभाग
[illegible] Psychiatry
[illegible] चिकित्सा शिक्षा एवं
[illegible] संस्थान, चण्डीगढ़ ।
PGIMER, Chandigarh

Dr. Sandeep Grover

Dr. Manmeet Kaur
Professor
Deptt. Of Comm. Medicine
PGIMER, Chandigarh

Dr. Arun Bansal
Professor
बाल रोग विज्ञान विभाग
Deptt. of Pediatrics
पी०जी०आई०, चण्डीगढ़-160 012
PGIMER, Chandigarh-160 012

Dr. Vikas Suri

Dr. Vikas Suri
Additional Professor
Department of Internal Medicine
PGIMER, Chandigarh-160012

Dr. Madhu Gupta
Professor
Deptt. Of Comm. Medicine
PGIMER, Chandigarh

Scanned with CamScanner

Minutes of the 3rd Doctoral Committee meeting

The 3rd Doctoral Committee meeting of Dr. Nimran Kaur, PhD student in the Department of Community, Medicine and School of Public Health was held on **29th September 2019 from 2:00 pm to 3:00 pm** in **Committee Room of Advanced Pediatric Centre** (Room number- 3105), PGIMER, Chandigarh. The session was chaired by Dr. Anil Bhalla, Professor, Advanced Pediatric Centre, PGIMER, Chandigarh. The following members were invited in the meeting:

1.	Dr. Anil Bhalla	Chairman
2.	Dr. Bhavneet Bharti	Member
3.	Dr. Manmeet Kaur	Member
4.	Dr. Vikas Suri	Member
5.	Dr. Arun Bansal	Member
6.	Dr. Manoj K. Rohit	Member
7.	Dr. Shankar Prinja	Member
8.	Dr Madhu Gupta	Chief guide
9.	Dr. Prahbhjot Malhi	Special invitee
10.	Dr. Sandeep Grover	Special invitee

कुल सचिव कार्यालय, पी.जी.आई.एम.ई.आर. चण्डीगढ़
Registrar Office PGIMER, Chandigarh
डायरी संख्या/[illegible]
TRG TRB006513
दिनांक/Date 13/9/19

Out of the total eight members, seven members were present in the meeting including Dr. Anil Bhalla (Professor, Advanced Pediatric Centre), Dr. Vikas Suri (Professor, Department of Internal Medicine), Dr. Arun Bansal (Professor, Advanced Pediatric Centre), Dr. Bhavneet Bharti (Professor, Advanced Pediatric Centre), and Dr. Madhu Gupta (Professor, School of Public Health & Department of Community Medicine). Dr Manmeet Kaur (Professor, School of Public Health & Department of Community Medicine) was out of station. Special invitees were Dr. Sandeep Grover (Professor, Department of Psychiatry) and Dr. Prahbhjot Malhi (Professor, Advanced Pediatric Centre) attended the meeting. Following are the minutes of the meeting:

1. It was suggested to mention the recall period of reporting screen-time by the caretaker in the methods section.
2. The primary outcome (screen-time) should be measured objectively by asking about the programs the children viewed before and after the intervention.
3. A lie detection test should be incorporated in the intervention implementation plan to remove the social desirability factor of the families and validating the data given by the families.
4. Developing videos for delivering the intervention:
   - 4.1. The content of the intervention program should be delivered with the help of videos as conducting so many house visits (440*8=3520) will not be feasible.
   - 4.2. The duration of the videos should not be more than 2 minutes per week, so total of 16-18 minutes video for the 8 week intervention should be developed.
   - 4.3. The videos need to be approved in an expert group meeting before disseminating them to the intervention group.

Scanned with CamScanner

4.4. The videos should be pretested in 20 families.

4.5. Some questions related to the videos should be asked on the weekly follow-up to make sure the videos were watched.

5. Delivering the intervention package:

5.1. The interventions may be given in batches to build a community dialogue and encourage peer pressure to make the intervention effective.

5.2. The first visit should be made by the researcher to build up a rapport and explain the videos in detail to the families. The subsequent visits may be done by the field investigators along with telephonic and social media follow-up with the families.

5.3. Any family that misses three sessions should be considered a drop out family.

6. The primary outcome (screen-time) should be measured every week in both the arms (intervention and control).

7. Analysis of the intervention should be done by both intention to treat and per protocol analysis to measure the effect of the intervention.

8. The committee applauded the results of the study and asserted that manuscripts should be written along with the work done.

Dr. Anil Bhalla
Dr. A.K. Bhalla
Professor
Deptt. of Pediatrics
PGIMER, Chandigarh

Dr. Sandeep Grover
Dr. Sandeep Grover
Assistant Professor
Deptt. of Psychiatry
P.G.I.M.E.R., Chandigarh
PGIMER, Chandigarh

Dr. Arun Bansal
Professor
बाल रोग विज्ञान विभाग
Deptt. of Pediatrics
पीजीआईएमईआर, चण्डीगढ़-160 012
PGIMER, Chandigarh-160 012

Dr. Vikas Suri
Dr. Vikas Suri
Additional Professor
Department of Internal Medicine
PGIMER, Chandigarh-160012

Dr. Bhavneet Bharti
आचार्य
Professor
बाल रोग विज्ञान विभाग
Deptt. of Pediatrics
पीजीआईएमईआर, चण्डीगढ़-160 012
PGIMER, Chandigarh-160 012

Dr .Shankar Prinja
अपर प्रोफेसर
Additional Professor
जन स्वास्थ्य स्कूल
School of Public Health
पी. जी. आई. एम. ई. आर., चण्डीगढ़
PGIMER, Chandigarh

Dr. Madhu Gupta
Professor
Deptt. Of Comm. Medicine
PGIMER, Chandigarh

Dr. Prahbhjot Malhi
आचार्य
Professor
बाल रोग विज्ञान विभाग
Deptt. of Pediatrics
पीजीआईएमईआर, चण्डीगढ़-160 012
PGIMER, Chandigarh-160 012

Scanned with CamScanner

Minutes of the 4th Doctoral Committee meeting

The 4th Doctoral Committee meeting of Dr. Nimran Kaur, PhD student enrolled under Dr. Madhu Gupta, Professor of Community Medicine in the Department of Community Medicine and School of Public Health was held on **14th October 2020** from **3:00 pm** to **4:00 pm** virtually on **Zoom**, at PGIMER, Chandigarh. The session was chaired by Dr. Anil Bhalla, Professor, Advanced Pediatric Centre, PGIMER, Chandigarh. DC members invited in the meeting were:

1.	Dr. Anil Bhalla	Chairman
2.	Dr. Bhavneet Bharti	Member
3.	Dr. Manmeet Kaur	Member
4.	Dr. Vikas Suri	Member
5.	Dr. Arun Bansal	Member
6.	Dr. Manoj K. Rohit	Member
7.	Dr. Shankar Prinja	Member
8.	Dr Madhu Gupta	Chief guide
9.	Dr. Prahbhjot Malhi	Special invitee
10.	Dr. Sandeep Grover	Special invitee

PGIMER, Chandigarh
TRB00.6170
दिनांक / Date 5/11/2020

Out of the total eight members, five members were present for the meeting including Dr. Anil Bhalla (Professor, Advanced Pediatric Centre), Dr. Vikas Suri (Professor, Department of Internal Medicine), Dr. Arun Bansal (Professor, Advanced Pediatric Centre), Dr. Manmeet Kaur (Professor, Department of Community Medicine & School of Public Health), and Dr. Madhu Gupta (Professor, School of Public Health & Department of Community Medicine). Dr. Prahbhjot Malhi (Professor, Advanced Pediatric Centre) was the special invitee.

Dr. Nimran Kaur presented the results of the study objective 2 on designing the intervention including the videos developed for the study participants; and objective 3 on baseline data collected for the intervention study. The following observations were made by the committee members:

1. The chairperson suggested that the introductory intervention video should be descriptive and of 5 to 8 minutes duration.

Scanned with CamScanner

2. Dr. Madhu Gupta proposed the Doctoral Committee to reduce the sample size of the intervention study from 440 to 340 (by reducing the attrition from the estimated 25% to 5%), as Dr. Nimran Kaur was finding it difficult to complete the sample size in the intervention study due to following reasons:
   a. Many families could not be contacted, as they had either shifted to their native places or were not no longer interested in continuing the intervention study.
   b. As the home-based intervention could not be delivered due to the Covid-19 lockdown after the baseline assessment (March 2020), the time left for PhD completion was less.
3. The Doctoral Committee agreed for reduction of sample size to 340 considering the pandemic situation.
4. It was told to complete the intervention study timely as it is 8 months long and only 9 months left to complete the Ph.D. program.

Dr. Anil Bhalla

प्राध्यापक
Professor
बाल रोग विज्ञान विभाग
Deptt. of Pediatrics
पीजीआई,चण्डीगढ़-160 012
PGIMER, Chandigarh-160 012

Dr. Arun Bansal

प्राध्यापक
Professor
बाल रोग विज्ञान विभाग
Deptt. of Pediatrics
पीजीआई,चण्डीगढ़-160 012
PGIMER, Chandigarh-160 012

Dr. Manmeet Kaur

आचार्य
Professor
[illegible]

Dr. Vikas Suri

Dr. Prahbhjot Malhi

प्राध्यापक
Professor
बाल रोग विज्ञान विभाग
Deptt. of Pediatrics
पीजीआई,चण्डीगढ़ [illegible]
PGIMER, Chandi[illegible]

Dr. Vikas Suri
Additional Professor
Department of Internal Medicine
PGIMER, Chandigarh-160012

Dr. Madhu Gupta

आचार्य
Professor
[illegible]
P.G.I.M.E.R.
Chandigarh

Scanned with CamScanner
